# Chronic isoprenaline/phenylephrine vs. exclusive isoprenaline stimulation in mice: critical contribution of alpha_1_-adrenoceptors to early cardiac stress responses

**DOI:** 10.1007/s00395-022-00920-z

**Published:** 2022-03-14

**Authors:** Matthias Dewenter, Jianyuan Pan, Laura Knödler, Niklas Tzschöckel, Julian Henrich, Julio Cordero, Gergana Dobreva, Susanne Lutz, Johannes Backs, Thomas Wieland, Christiane Vettel

**Affiliations:** 1 Institute of Experimental Cardiology, Internal Medicine VIII, Heidelberg University, Heidelberg, Germany; 2 DZHK (German Center of Cardiovascular Research), Partner Site Heidelberg/Mannheim and Göttingen, Germany,; 3 Experimental Pharmacology Mannheim, European Center for Angioscience, Medical Faculty Mannheim, Heidelberg University, Mannheim, Germany Ludolf-Krehlstr. 13-17, 68167; 4 Division of Life Sciences and Medicine, Department of Cardiology, The First Affiliated Hospital of USTC, University of Science and Technology of China, Hefei, 230001 Anhui China; 5 Experimental Cardiology, European Center for Angioscience, Medical Faculty Mannheim, Heidelberg University, Mannheim, Germany; 6 Institute of Pharmacology and Toxicology, University Medical Center Göttingen, Göttingen, Germany

**Keywords:** Neurohumoral models of cardiac remodeling, Isoprenaline, Phenylephrine, α_1_- and β-adrenoceptors, α_1_-adrenergic inotropy, Cardiac fibroblast activation

## Abstract

Hyperactivity of the sympathetic nervous system is a major driver of cardiac remodeling, exerting its effects through both α-, and β-adrenoceptors (α-, β-ARs). As the relative contribution of subtype α_1_-AR to cardiac stress responses remains poorly investigated, we subjected mice to either subcutaneous perfusion with the β-AR agonist isoprenaline (ISO, 30 mg/kg × day) or to a combination of ISO and the stable α_1_-AR agonist phenylephrine (ISO/PE, 30 mg/kg × day each). Telemetry analysis revealed similar hemodynamic responses under both ISO and ISO/PE treatment i.e., permanently increased heart rates and only transient decreases in mean blood pressure during the first 24 h. Echocardiography and single cell analysis after 1 week of exposure showed that ISO/PE-, but not ISO-treated animals established α_1_-AR-mediated inotropic responsiveness to acute adrenergic stimulation. Morphologically, additional PE perfusion limited concentric cardiomyocyte growth and enhanced cardiac collagen deposition during 7 days of treatment. Time-course analysis demonstrated a diverging development in transcriptional patterns at day 4 of treatment i.e., increased expression of selected marker genes *Xirp2, Nppa, Tgfb1, Col1a1, Postn* under chronic ISO/PE treatment which was either less pronounced or absent in the ISO group. Transcriptome analyses at day 4 via RNA sequencing demonstrated that additional PE treatment caused a marked upregulation of genes allocated to extracellular matrix and fiber organization along with a more pronounced downregulation of genes involved in metabolic processes, muscle adaptation and cardiac electrophysiology. Consistently, transcriptome changes under ISO/PE challenge more effectively recapitulated early transcriptional alterations in pressure overload-induced experimental heart failure and in human hypertrophic cardiomyopathy.

**Supplementary Information:**

The online version contains supplementary material available at 10.1007/s00395-022-00920-z.

## Introduction

Hyperactivity of the sympathetic nervous system (SNS) is a central feature of heart failure (HF). The SNS-mediated increase in contraction force and heart rate is exerted through the release of the α/β-adrenoceptor (AR) agonists noradrenaline and adrenaline from sympathetic neurons and the adrenal glands. Its activation is a compensatory mechanism that temporarily maintains cardiac output and thus sufficient organ perfusion and tissue oxygen supply. However, persistent AR stimulation promotes molecular and structural changes including pathological hypertrophy [69], cardiac fibrosis [2], electrophysiological and metabolic alterations [7, 19], and inflammation [45] with deteriorating effects on cardiac function. In addition, SNS hyperactivity evokes neurohumoral responses such as the activation of the renin angiotensin aldosterone system (RAAS), another critical contributor to pathological remodeling processes inflicted on the heart [23].

α-ARs and β-ARs are expressed in several subtypes throughout the cardiovascular system. In the heart, the most prominent AR is the subtype β_1_. It is exclusively expressed in cardiomyocytes and the main mediator of the cardiac response to changes in the sympathetic tone. During chronic adrenergic stress, β_1_-AR signaling is subject to different desensitization mechanisms, which involve not only receptor downregulation, but also intracellular adaptations regarding abundance and activity of effectors and regulators within the β_1_-AR signaling cascades [14, 48]. In contrast, the β_2_-AR subtype is expressed in both cardiomyocytes and non-cardiomyocytes [46]. In cardiac fibroblasts, β_2_-AR signaling promotes proliferation and interleukin-6 release [65], the latter a well-known mediator of the fibroblast/cardiomyocyte crosstalk involved in cardiac hypertrophy [37]. However, β_2_-ARs and especially their downstream effector cAMP were also reported to block fibroblasts to myofibroblast transition in cell culture models e.g., by downregulating the expression of α-smooth muscle actin, collagen, and connective tissue growth factor [10, 61, 71]. Finally, the third subtype of β-ARs, β_3_-AR, has been found expressed at low levels in cardiomyocytes and in cardiac endothelial cells, where it is involved in the production of nitric oxide. Its enhanced activity during HF is therefore viewed as cardioprotective [42].

Cardiac α_1_-AR receptors are expressed in three subtypes, α_1A_, α_1B_, and α_1D_, with α_1A_ restricted to a subpopulation of cardiomyocytes [46], and α_1D_ to smooth muscle cells along with postsynaptic α_2_-ARs [25, 26, 29]. In contrast to β_1_-ARs, substantial evidence points to a protective role of at least the α_1A_-subtype in cardiac diseases [12, 13, 78]. α_1_-ARs have been described as mediators of physiological hypertrophy [51], although their involvement remains controversial [31], and as an essential component of cardiac contractility especially during β_1_-AR desensitization [28, 58]. However, α_1_-ARs are G_q_-protein-coupled receptors and therefore associated with the activation of transcriptional pathways shared by other GPCRs like the angiotensin II type 1 receptor (AT_1_R) and endothelin-1 receptors [40, 69]. Consistently, α_1_-ARs are potent inducers of hypertrophic growth and fetal gene expression in cell-based systems such as neonatal rat cardiomyocytes or engineered heart tissue and their activation is commonly used to explore the respective stress-related pathways [34].

Apart from smooth muscle cells, there is little evidence of a functional α_1_-AR expression in non-cardiomyocytes, including fibroblasts in the heart [61, 73]. Nonetheless, chronic perfusion with angiotensin II (Ang II) is now increasingly combined with the application of the stable α_1_-AR agonist phenylephrine (PE) to aggravate stress responses and cytokine release in models focusing on pro-fibrotic processes [9, 18, 32]. In preclinical models where heart failure is surgically induced e.g., by coronary vessel ligation or transversal aortic constriction (TAC), endogenous activation of the SNS leads to the stimulation of both α_1_- and β-ARs. However, regarding pharmacologically induced adrenergic stress, the most widely used approach remains either bolus injections or chronic infusion of the β-AR agonist isoprenaline (ISO) [5]. In contrast, combinations of ISO and PE are rarely used as a pharmacological heart failure model and if so, often focused on α_1_-AR/G_q_-dependent impact on crosslinking dynamics during the contraction cycle or on the expression and posttranslational modifications of proteins involved in this process [3, 20, 56]. As the combined perfusion of ISO and PE in mice is assumed to resemble more the pathophysiological situation of sympathetic overdrive in the development of human heart failure, we intended with this explorative study to characterize the ISO/PE model in more detail by directly comparing the outcome on hemodynamics, cardiac performance, cardiac morphology and cardiac gene expression changes to exclusive ISO exposure. Our data show that additional chronic PE stimulation is an essential stimulus to re-establish α_1_-AR-mediated inotropic responsiveness to acute adrenergic stress during β_1_-AR desensitization, and that chronic ISO/PE stimulation compared with exclusive ISO stimulation more effectively recapitulates early transcriptome alterations induced by pressure overload models in mice and most importantly, human hypertrophic cardiomyopathy.

## Experimental procedures

### Animals

All animal experiments were carried out according to the European Community guiding principles of care and use of animals (2010/63/UE, 22 September 2010). Authorizations were obtained from Regierungspräsidium Karlsruhe, Germany (AZ-G47/18). The mice used for this study were on a pure C57/Bl6N background and either bred in the animal facility of Heidelberg University (IBF) or in case of the losartan and IonOptix experiments purchased from Janvier Labs. For the experiments only mice with a C57/Bl6N background at the age of 3–4 months were included and separated into sex and age matched groups.

### Chronic isoprenaline/phenyephrine administration

Isoprenaline and phenyephrine (ISO and PE, Sigma-Aldrich) were delivered to mice by subcutaneously implanted osmotic minipumps (Alzet, model 1007D) that released ISO or ISO and PE dissolved in 0.9% NaCl at a dose of 30 mg/kg × day each. The chosen dose represents a commonly used treatment regarding ISO models [5] and is a validated subcutaneously applied pressor dose of PE [30]. Anesthesia was performed with isoflurane (1.5% v/v) and mice were provided with the analgesic carprofen (CP-Pharma, 4 mg/kg, s.c.) prior to the skin incision. The wound was closed using a non-absorbable surgical threat (Ethicon Prolene). Animals were sacrificed by cervical dislocation at the indicated time points.

### Echocardiography

Echocardiographic analysis was performed after 1 day and 6 days of chronic catecholamine perfusion at basal conditions and after the injection of dobutamine (Fresenius, 10 mg/kg i.p.). Animals were kept under light temperature-, respiration- and ECG-controlled anesthesia (isoflurane, 1–1.5% v/v) during the whole procedure. Echocardiography was performed on a Vevo 2100^®^ System (Visual Sonics Inc.) equipped with a MS400 transducer. B-Mode and M-Mode images were obtained in parasternal long axis and in short axis view at midpapillary muscle level. To determine % fractional shortening, 5 contraction cycles of short axis M-Mode recordings were analyzed with the VevoLab software using the LV trace tool.

### Isolation of adult mouse ventricular myocytes

For the isolation of adult cardiomyocytes, mice were euthanized by cervical dislocation. The heart was carefully removed and transferred into a 6 cm dish containing ice cold perfusion buffer (in mM: NaCl 113, KCl 4.7, KH_2_PO_4_ 0.6, Na_2_HPO_4_ 0.6, MgSO_4_ 1.2, NaHCO_3_ 12, KHCO_3_ 10, HEPES 10, Taurine 30, Glucose 5.5, BDM 10, pH 7.4 adjusted with KOH). The aorta was then clamped to a cannula and fixed with 6–0 surgical silk, followed by perfusion with perfusion buffer at 37 °C for 2 min. For enzymatic dissociation, the heart was perfused with digestion buffer (perfusion buffer with 12.5 µM Ca^2+^, containing liberase (Sigma-Aldrich) and trypsin (Thermo Fisher)) at a flow rate of 3.5 ml/min for 13 min. Then the heart was placed into a dish containing 2.5 ml digestion buffer, cut into small pieces and the tissue was gently pipetted up and down using a fine-tip transfer pipette to disperse the large pieces. 2.5 ml stop buffer 1 (perfusion buffer with 50 µM Ca^2+^ and 1% BSA (Sigma-Aldrich)) were added and ventricular myocytes were filtered through a mesh (200 µm) and allowed to sediment by gravity for 11 min. Thereafter, the supernatant was discarded, 5 ml stop buffer 2 (perfusion buffer with 38 µM Ca^2+^ and 0.5% BSA) were added and the Ca^2+^ concentration was stepwise increased to 1 mM.

### Ca^2+^ measurements and fractional shortening

Isolated cardiomyocytes were seeded on laminin-coated glass-bottomed dishes, and let attach for two hours before incubation with Fura-2 AM (1 μM) in Tyrode’s buffer (in mM: KCl 4, NaCl 140, MgCl_2_ 1, HEPES 5, Glucose 10, and 1 CaCl_2_ 1) for 20 min (37 °C, 5% CO_2_). Thereafter, the cells were carefully washed with fresh Tyrode’s buffer to remove the remaining dye. Ca^2+^ transients and sarcomere shortening were recorded on an IonOptix setup using the IonWizard software according to the manufacturer’s instructions. Cells were paced electrically with 10 V and at a pacing frequency of 1 Hz. Fura-2/Ca^2+^ transients were captured at alternating wavelengths 340/380 nm, and emission was recorded at 510 nm. Sarcomere shortening was analyzed by Fourier transform analysis of the cardiomyocyte striations under phase-contrast microscopy. To differentially stimulate α_1_- and β-adrenoceptors, cardiomyocytes were pre-incubated with either α_1_-adrenergic antagonist prazosin (10 μM, Sigma-Aldrich) or β-adrenergic anatagonist atenolol (1 μM, TCI) for 5 min and then stimulated with the pan-adrenergic agonist dobutamine (2.5 μM). All measurements were carried out at 37 ± 1 °C.

### Histological analysis

Hearts were fixed in 4% (m/v) paraformaldehyde, embedded in paraffin, and sliced into transversal sections (5 µm). To determine the cross-sectional area of cardiomyocytes, the tissue was rehydrated, incubated with Lectin-WGA-TRITC (Sigma-Aldrich) over night (4 °C), and covered with ROTI^®^Mount FluorCare (Carl Roth). To document collagen deposition, slides were rehydrated and incubated for 1 h (RT) with Picro/Sirius Red (Niepötter Labortechnik) solution, and covered with Entellan^®^ (Sigma-Aldrich). Slides were scanned (Axioscan.Z1, Zeiss) and analyzed with ImageJ software. For cross-sectional area determination, 117–675 randomly chosen transversely cut cardiomyocytes per individual were analyzed. To analyze collagen deposition, the Picro/Sirius Red-stained area of whole transversal sections was quantified. Analyses were performed blinded to group allocation.

### ECG and blood pressure telemetry

Mice were anaesthetized with isoflurane (2.5% v/v) via mask ventilation and placed on a warming plate (37 °C). The skin of the anterior neck thoracic region was depilated and disinfected, and a 2.5 cm long median incision of the skin was made. The underlying tissue was prepared for fixation of the electrodes, the submaxillary glands were gently separated, and the left common carotid artery was isolated. The carotid artery was cannulated, and the catheter of the transmitter (HD-X11, DSI) was advanced into the carotid artery, so that the tip of the catheter was placed in the aortic arch, and fixed with 3 silk ligatures (Ethicon). The negative electrode of the transmitter was fixed to the right pectoralis fascia and the positive electrode was fixed 1 cm left to the xiphoid. The transmitter body was placed into a subcutaneous pocket along the left flank of the mouse. The wound was closed with using 6–0 Prolene (Ethicon). Carprofen (CP-Pharma, 4 µg/g, s.c.) was applied once before starting the surgical procedure and once daily up to 48 h after the surgical procedure for intra- and postoperative analgesia. Recordings were started after a recovery time of two weeks post subcutaneous implantation of the telemetric transmitter. Recording and analysis parameters were set according to the manufacturer’s instructions using Ponemah P3 Plus software (DSI). Heart rate and blood pressure values are presented as averages of 1 min or of 5 s intervals. Details are specified in the respective figure legends.

### Tail cuff blood pressure measurement and losartan treatment

Non-invasive blood pressure measurements were performed on a Coda2 tail cuff system (Kent Scientific). Mice were placed into rodent holders on a warming platform 10 min prior to obtaining the measurements. At least 5 replicate blood pressure values were measured to calculate a mean for each individual. Losartan was administered via drinking water (600 mg/l) [77]. Treatment was started 1 day before minipump implantation and continued throughout the ISO or ISO/PE perfusion period.

### RNA-isolation

Pulverized right and left ventricular tissue was dissolved in TRIzol^™^ reagent (Thermo Fisher) and RNA extraction was performed according to the manufacturer’s instructions.

### Quantitative PCR

First-strand cDNA synthesis was performed with SuperScript IV VILO Master Mix (Thermo Fisher) and the 1:5 diluted product was added to the TaqMan^™^ Fast Advanced Mastermix (Thermo Fisher) according to the manufacturer’s instructions. The following probes (Thermo Fisher) were applied to quantify gene expression: Acta1 (Mm00808218_g1), Adra1a (Mm00442668_m1), Adra1b (Mm00431685_m1), Adra1d (Mm01328600_m1), Adrb1 (Mm00431701_s1), Adrb2 (Mm02524224_s1), Adrb3 (Mm02601819_g1), Col1a1 (Mm00801666_g1), Myh7 (Mm00600555_m1), Nppa (Mm01255747_g1), Nppb (Mm01255770_g1), Nr4a1 (Mm01300401_m1), Postn (Mm01284919_m1), Ppia (Mm02342430_g1), Rcan1 (Mm01213406_m1), Xirp2 (Mm01335343_m1). Real-time PCR was performed with QuantStudio3 (Thermo Fisher) and analyzed based on ΔΔCT calculations using peptidylprolyl isomerase A (Ppia) as reference gene [41, 44].

### Transcriptome analysis

RNA-Seq reads were mapped to the mm10 reference genome from UCSC using STAR (-alignIntronMin 20 -alignIntronMax 500,000). Samples were quantified using analyzeRepeats.pl (mm10 -count exons -strand both -noadj). Differential expression was quantified and normalized using DESEq2. Reads per kilobase per millions mapped (rpkm) was determined using rpkm.default from EdgeR. The PCA plots in each RNA-Seq dataset were obtained using rowVars and prcomp into a custom R-script. Genes with an FDR adjusted p value (q value) of < 0.05 and a log2fold change of > 0.6 or < − 0.6 were considered significantly regulated (accession number GSE195466). The overlap of regulated genes was visualized with BioVenn [27], and the resulting gene lists were then subjected to enrichment analyses using Metascape limited to the GO domain “biological processes” [81]. For the visualization of heat maps, RPMK values of either manually curated gene lists generated from the indicated enriched pathways or all genes significantly regulated under ISO/PE were hierarchically clustered using Morpheus software (https://software.broadinstitute.org/morpheus) or converted into log2fold changes for the comparison with the TAC data set. RPKM of selected marker genes were visualized with Integrated Genome Viewer (Suppl. Figs. 6, 7) [66].

### Selection of TAC and HCM bulk RNAseq data sets for comparison

The literature was searched for published, accessible, and high-quality data sets obtained from mice and humans with an anticipated similar stage-specific transcriptional profile i.e., early phase post transaortic constriction [49] and patients diagnosed with hypertrophic cardiomyopathy, but not end-stage heart failure [54]. For comparison, the transcriptional background of ISO and ISO/PE samples was adjusted to genes present in the respective human or TAC data set and vice versa.

### Illustrations

Illustrations were generated with Biorender software or downloaded from Smart Servier Medical Art (https://smart.servier.com) and adapted with adobe illustrator version 25.3.1.

### Statistics

Statistical analyses were performed with GraphPad prism 9. Results are presented as mean ± SD or median ± IQR for non-Gaussian distributed data. Data sets were tested for normal distribution (Anderson–Darling, D’Agostino-Pearson omnibus, Shapiro–Wilk, Kolmogorov-Smirnovand), and compared by unpaired *t* test, 1-way ANOVA, 2-way ANOVA, Brown-Forsythe and Welch ANOVA, or Kuskal–Wallis test followed by multiple comparison testing. For each data set the respective statistical test is indicated in the figure legend. *P* values are presented within each figure. Effect sizes were calculated as difference of two means divided by the pooled SD (Cohen’s *d*). Unequal *n* numbers were taken into account for pooled SD calculations.

## Results

### Similar hemodynamic profile and RAAS activation in ISO and ISO/PE-treated mice

**Fig. 1:**
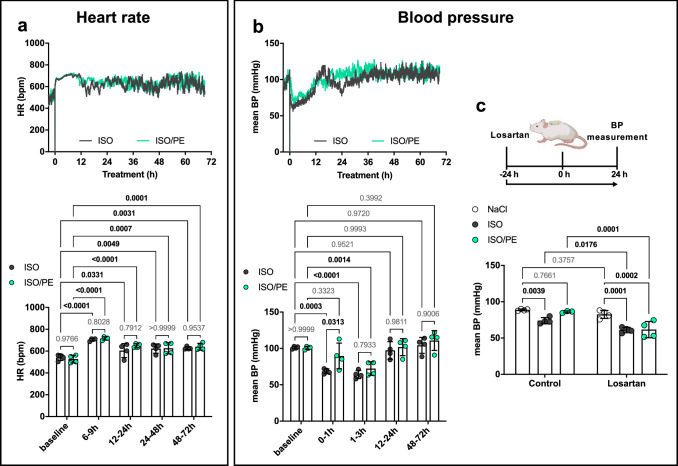
Hemodynamic profile of isoprenaline (ISO) or isoprenaline/phenylephrine (ISO/PE) treated mice before and during the first 72 h after osmotic minipump implantation. **a** Heart rate (HR) and **b** mean blood pressure (BP) were monitored by ECG and blood pressure telemetry in freely moving mice. Calculations of average values for HR and BP at the indicated time points are given in the lower panels including an average of 3 d baseline recordings. Individual data, mean ± SD of n = 4 animals (2 males/2 females per group) are shown. *P* values were determined by repeated measures 2-way ANOVA followed by Dunnett’s multiple comparison test (vs. baseline) or by Sidak`s multiple comparison test (ISO vs. ISO/PE for each time point). **c** Mean blood pressure was measured by tail cuff in restrained mice after 24 h of either NaCl, ISO or ISO/PE treatment, in the presence or absence of AT_1_R antagonist losartan (treatment was started 24 h before minipump implantation). Individual data, mean ± SD of *n* = 4 males per group are shown. *P* values were determined by 2-way ANOVA followed by Dunnett’s multiple comparison test (vs. NaCl) or by Sidak’s multiple comparison test (vs. losartan)

To investigate how additional PE stimulation affects hemodynamic parameters, heart rate (HR) and blood pressure (BP) were monitored via implanted telemetry transmitters in mice perfused with either 30 mg/kg × day ISO or 30 mg/kg × day ISO plus 30 mg/kg × day PE (ISO/PE). Both treatments induced a strong increase in HR that reached a maximum > 700 bpm between 6–9 h post minipump implantation and a decrease in mean BP from 100 to 70 mmHg (Fig. 1a, b). Apart from a delayed decrease in mean BP during the first hour post implantation, PE showed no persistent vasoconstrictive or baroreflex effects in the presence of ISO and hence did not antagonize ISO-induced BP and HR regulation even though it was applied in a validated pressor dose [30]. Instead, vasodilation and positive chronotropic effects were the dominating hemodynamic factors in both groups. Similar results were obtained after acute injections of ISO/PE (Suppl. Fig. 1c). In both chronic perfusion models, HR stabilized at 625 bpm after 12–24 h, and thus remained permanently higher than at baseline (Fig. 1a, lower panel). In contrast, mean BP slowly recovered over the first 24 h and returned to pre-treatment levels (Fig. 1b) with a minor elevation at later time points (144–168 h, ΔmeanBP approx. 10 mmHG, Suppl. 2d). Since these results indicated an increase in RAAS activity, effects on BP were analyzed in a follow-up experiment by applying the AT_1_R antagonist losartan. Indeed, AT_1_R blockade prevented BP stabilization in ISO and ISO/PE-treated animals, suggesting an increase in Ang II levels as an additional factor in both models (Fig. 1c).

### Chronic α_1_-AR stimulation promotes contractile responsiveness to acute adrenergic stress during β_1_-adrenergic desensitization

**Fig. 2:**
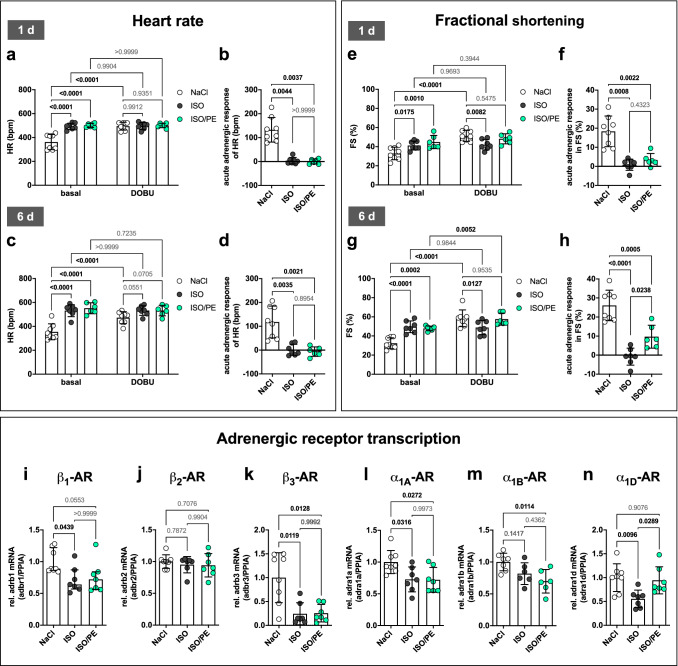
Echocardiography analysis of cardiac function and cardiac adrenoreceptor (AR) expression levels in control (NaCl), isoprenaline (ISO) and isoprenaline/phenylephrine (ISO/PE) treated mice. **a–d** Heart rate (HR), and **e–h** percent fractional shortening (% FS) after 1 d (**a, b, e, f**) or 6 d (**c, d, g, h**) of chronic adrenergic stress. After the assessment of basal parameters, animals were challenged with 10 mg/kg of the pan-adrenergic agonist dobutamine (DOBU, i.p.) to measure acute adrenergic responsiveness. All functional parameters were recorded under anesthesia (1% v/v isoflurane). **i–n** mRNA levels of the different adrenoceptor subtypes (AR) analyzed by qPCR in total heart tissue 7 d after osmotic minipump implantation. Data was normalized to the expression levels of peptidylprolyl isomerase A (Ppia) and are given relative to control (NaCl). **a–n** Individual data, mean ± SD except **b, i** median ± IQR of NaCl *n* = 8 (4 males/4 females), ISO *n* = 7 (4 males/3 females) and ISO/PE *n* = 6–7 (**a–h** 3 males/3 females and **i–n** 4 males/3 females). *P* values were determined by **a c, e, g** repeated measures 2-way ANOVA followed by Dunnett`s multiple comparison test (vs. NaCl) or by Sidak’s multiple comparison test (vs. DOBU), **b, i** Kruskal–Wallis followed by Dunn’s multiple comparisons test, **d, f, k** Brown–Forsythe and Welch ANOVA Dunnett T3 Multiple Comparison, **h–j, l–n** ordinary 1-way ANOVA followed by Tukey’s multiple comparison test

Acute α_1_-AR stimulation was shown to contribute to adrenergic driven increase in contractility in isolated cardiac muscle stripes of both rodents and humans [16], and in α_1A_-AR overexpressing transgenic mice [6, 43, 79]. To assess the impact of endogenous α_1_-AR activity on cardiac inotropy and adrenergic desensitization during ISO/PE challenge, mice were subjected to echocardiography on day 1 and day 6 after implantation of the respective osmotic minipumps. Both ISO and ISO/PE-treated mice showed higher basal heart rates at day 1 and 6 compared to the control group. The heart rate elevation reflected the maximal response of control animals under acute adrenergic stimulation with the AR agonist dobutamine (DOBU 10 mg/kg), and no responsiveness to additional DOBU stimulation could be detected in the ISO and ISO/PE groups (Fig. 2a–d). Both ISO and ISO/PE-treated animals showed higher basal contraction force compared to control animals (Fig. 2e, g). Neither ISO nor ISO/PE animals were responsive to acute adrenergic stimulation 1 d after the onset of catecholamine treatment, and thus did not reach the maximal response evoked by DOBU in control animals (Fig. 2e, f). However, after 6 d, ISO/PE-treated mice showed an increase of 10% (effect size *d* = 2.0 vs. ISO) in fractional shortening after challenged with DOBU (Fig. 2g, h). This responsiveness to acute adrenergic stress was absent in ISO-treated animals and independent of transcriptional AR regulation as similar reductions in β_1_-, β_3_-, α_1A_- and α_1B_-AR mRNA were observed under both ISO and ISO/PE stimulation. Notably, the smooth muscle subtype α_1D_-AR was only downregulated under ISO, but not ISO/PE treatment (Fig. 2i–n). Thus, our data suggest that adrenergic responsiveness during β_1_-AR desensitization requires adaptations that are dependent on chronic α_1_-AR stimulation.

### Acute adrenergic responsiveness is mediated by α_1_-AR activation in ISO/PE-treated animals

**Fig. 3:**
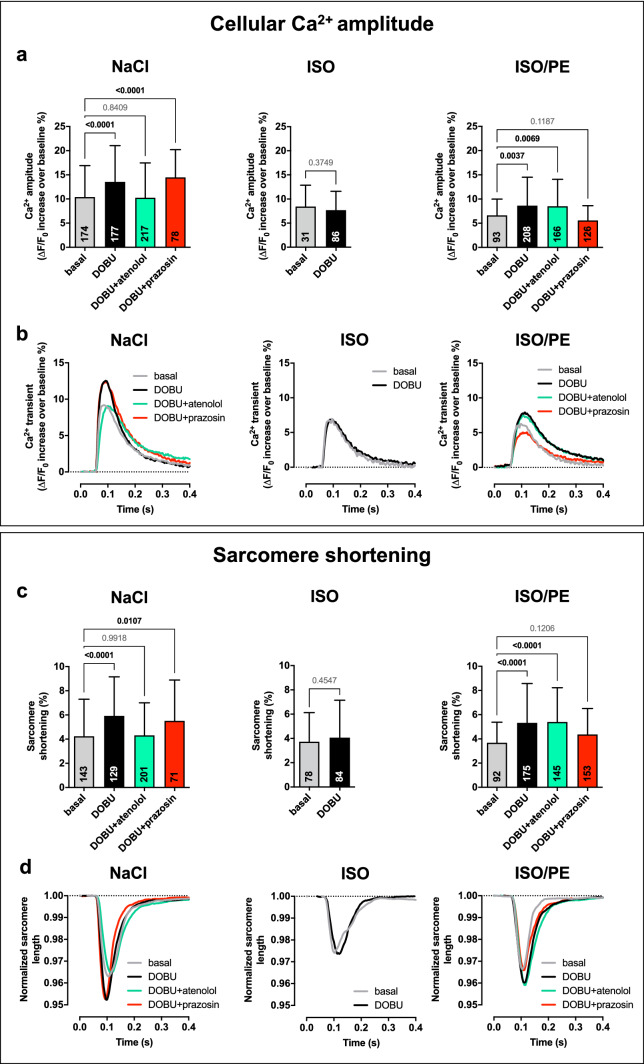
Measurement of Ca^2+^-transients and sarcomere shortening of ventricular cardiomyocytes isolated 6 d after osmotic minipump implantation from either control (NaCl), isoprenaline (ISO) or isoprenaline/phenylephrine (ISO/PE) treated mice. Cells were loaded with Fura-2 and stimulated at a frequency of 1 Hz in the absence or presence of 2.5 µM dobutamine (DOBU), and if indicated β-adrenoceptor antagonist atenolol (1 µM) or α_1_-adrenoceptor antagonist prazosin (10 µM). Sarcomere length and Fura-2 ratios were recorded using an IonOptix System. **a** Peak height, and **b** average of Ca^2+^- amplitudes given as percent increase over diastolic Fura-2 ratios. **c** Maximal sarcomere shortening, and **d** average of sarcomere lengths during contraction given relative to resting state. **a, c** Mean ± SD of NaCl *n* = 2–3 males, ISO *n* = 2 males, and ISO/PE *n* = 3 males, measured cell numbers per condition are indicated in the respective bar graph. *P* values were determined by ordinary 1-way ANOVA followed by Dunnett’s multiple comparison test vs. basal (NaCl and ISO/PE) or by unpaired *t* test vs. basal (ISO)

The adrenergic agonist DOBU, if used as a racemate, shows affinity to both α_1_-AR and β-ARs [75]. Therefore, we analyzed the origin of the responsiveness to DOBU-mediated Ca^2+^ release and sarcomere shortening in isolated cardiomyocytes in the presence of the β-AR antagonist atenolol and the α_1_-AR antagonist prazosin. To ensure effective receptor binding of the competitive antagonists, we used a concentration of the agonist DOBU in the range of its described EC_50_ (2.5 µM) [59] to document α_1_-AR vs. β-AR dependency of the inotropic response. In line with the in vivo results, cardiomyocytes from animals chronically treated with ISO remained unresponsive to DOBU, whereas cardiomyocytes isolated from the ISO/PE group showed higher Ca^2+^-amplitudes (Fig. 3a, b) and increased sarcomere shortening after DOBU challenge (Fig. 3c, d). In contrast to control animals, the effect was abolished in the presence of prazosin but not atenolol. These results are in line with earlier observations of an increased α_1_-AR-mediated inotropy during β_1_-AR desensitization demonstrated in a rat model of congestive HF [58]. However, our results additionally indicate that an efficient compensation requires structural and/or molecular changes that are mediated by chronic α_1_-AR stimulation.

### Limited concentric growth and enhanced collagen deposition in hearts of ISO/PE-treated animals

**Fig. 4:**
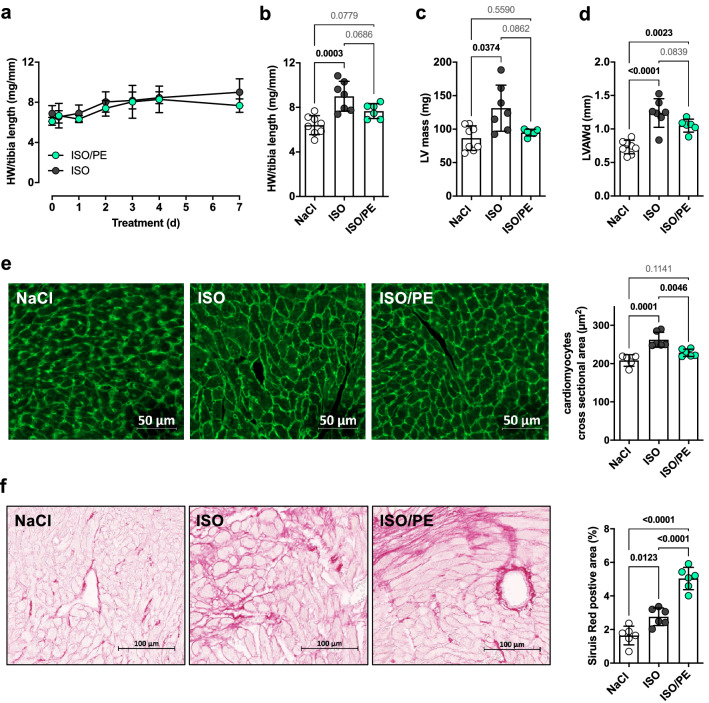
Assessment of cardiac hypertrophic growth and cardiac collagen deposition in either control (NaCl), isoprenaline (ISO) or isoprenaline/phenylephrine (ISO/PE) treated mice. **a, b** Ratio of heart weight to tibia length taken at the indicated time points. Mean ± SD of 0–4 d *n* = 2 males/2 females per time point and treatment, and 7 d *n* = 4 males/4 females (NaCl), 4 males/3 females (ISO), and 3 males/3 females (ISO/PE). **c, d** Left ventricular mass (LVM) and left ventricular diastolic anterior wall thickness (LVAWd) measured by echocardiography after 6 d of treatment *n* = 4 males/4 females (NaCl), 4 males/3 females (ISO), and 3 males/3 females (ISO/PE). **e** Representative images of WGA-stained cross sections from paraffin-embedded ventricular heart tissue taken 7 d after osmotic minipump implantation and quantification of cardiomyocyte cross-sectional area given as average of 117–675 cells per individual. Individual data, mean ± SD of *n* = 3 males/2 females (NaCl), and 3 males/3 females (ISO and ISO/PE). **f** Representative Sirius Red-stained cross sections from paraffin-embedded ventricular heart tissue taken 7 d after osmotic minipump implantation to indicate collagen deposition. Individual data, mean ± SD of *n* = 3 males/3 females per group. Values are given as percentage of total ventricle area. **b, d, e, f** *P* values were determined by one-way ANOVA followed by Tukey’s multiple comparison test or **c** Brown-Forsythe and Welch ANOVA Dunnett T3 Multiple Comparison

Next, we analyzed cardiac morphology in the different treatment groups. α_1_-AR activity has been reported to limit hypertrophy and cardiac dysfunction after myocardial infarction and pressure overload in α_1A_-AR overexpressing transgenic mice [12, 13]. To document the dynamics of hypertrophic growth, heart weight (HW) and tibia length (TL) were monitored between 0 and 4 d and at 7 d (Fig. 4a, b). Animals from both groups showed a similar progressive increase in HW/TL ratios during the first 4 days of adrenergic perfusion. After 4 days, hypertrophic growth appeared to be terminated in ISO/PE animals, whereas the heart weights of the ISO-treated group were still moderately increasing until day 7. The limited hypertrophic growth in the ISO/PE model was reflected in HW/TL ratios (effect size *d* = 1.3 vs. ISO), and LV mass (effect size *d* = 1.4 vs. ISO) and anterior wall thickness measured by echocardiography (effect size *d* = 1.1 vs. ISO; Fig. 4c, d). Histological analysis revealed that concentric growth of cardiomyocytes assessed as the average cross-sectional area was indeed markedly lower in ISO/PE-treated animals (effect size *d* = 2.2 vs. ISO; Fig. 4e). However, this limiting effect on cardiomyocyte growth was accompanied by a more prominent collagen deposition at day 7 (effect size *d* = 3.8 vs. ISO), indicating the increased presence of activated fibroblasts (Fig. 4f). Taken together, the morphological data suggest that myocardial stress response started to diverge at around 4 days after drug application and resulted in enhanced cardiomyocyte hypertrophy in the ISO model and early signs of fibrosis in the ISO/PE model.

### 4 d—a critical time point in α1-AR-mediated transcriptional effects

**Fig. 5:**
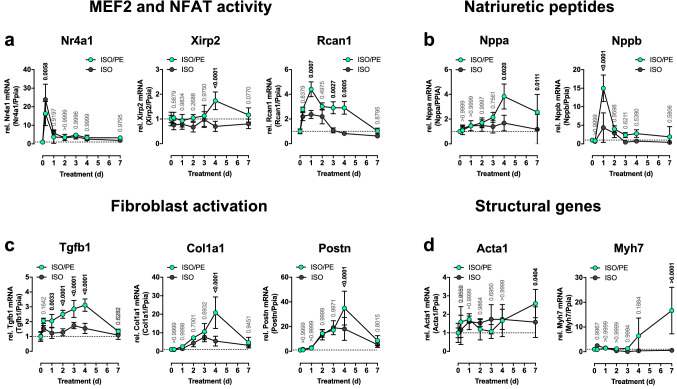
Transcriptional dynamics in cardiac tissue of the indicated marker genes during the first 7 d after isoprenaline (ISO) or isoprenaline/phenylephrine (ISO/PE) application. Values were normalized to the expression levels of peptidylprolyl isomerase A (Ppia) and are given relative to control (0 d or NaCl for 7 d). Mean ± SD, from *n* = 2 males/2 females per group and time point (0–4 d), and 4 males/3 females (7d). *P* values were determined by 2-way ANOVA followed by Sidak’s multiple comparison test (ISO vs. ISO/PE)

To analyze the transcriptional dynamics preceding the morphological changes, we monitored ventricular mRNA levels of selected marker genes over the course of the first 7 days. Target genes of key transcriptional factors such as MEF2 (*Nr4a1*, *Xirp2*) [38, 67], and NFAT (*Rcan1*) [70] (Fig. 5a) were included as well as the natriuretic peptides *Nppa* (ANP) and *Nppb* (BNP, Fig. 5b), genes associated with fibroblast activation (*Col1a1*, *Tgfb1*, *Postn,* Fig. 5c), and fetal structural genes such as *Acta1* and *Myh7* (Fig. 5d). All genes were normalized to the reference gene *Ppia,* of which the stable expression during the time course experiments is demonstrated in Suppl. Fig. 3. With the exception of *Nr4a1*, which is known to be sensitive to β-AR stimulation [47], the upregulation of all other analyzed genes was either more pronounced under ISO/PE (*Rcan1*, *Nppb*, *Postn, Col1a1*) including the pro-fibrotic cytokine *Tgfb1* [36] (effect sizes for peak expression *d* = 2.1, 2.6, 1.5, 2.4, 4.1 vs. ISO, respectively), or completely dependent on additional PE stimulation (*Xirp2*, *Nppa*, *Acta1*, *Myh7*, effect sizes for peak expression *d* = 3.5, 2.6, 1.3, 2.4 vs. ISO, respectively). Of note, most of the genes analyzed showed a transient upregulation with a peak expression at 4 d under ISO/PE conditions (*Xirp2, Nppa, Tgfb1, Col1a1, Postn*). In contrast, differential expression of structural genes such as *Acta1* and *Myh7* was observed later at 7 d. Collectively the data on both gene regulation and hypertrophic growth suggest that 4 d is a critical turning point for differential morphological and transcriptional responses in the ISO vs. ISO/PE model. Hence, this time point was chosen for a subsequent transcriptome analysis.

### ISO/PE promotes the upregulation of genes associated with ECM organization and interaction

**Fig. 6:**
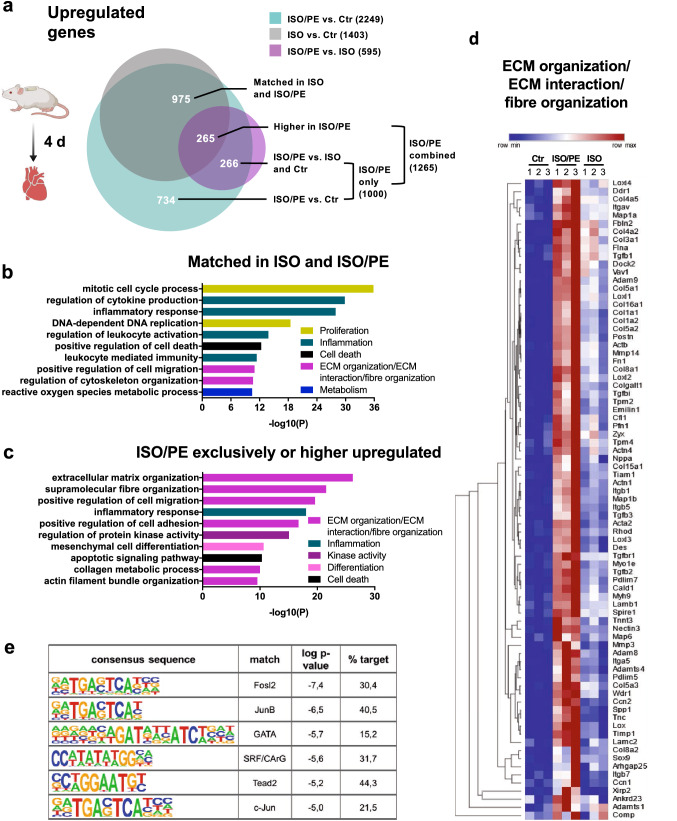
Upregulated genes (log2FC > 0.6, *q* value < 0.05) assessed by bulk RNAseq analysis of cardiac tissue from control (0 d), isoprenaline (ISO) and isoprenaline/phenylephrine (ISO/PE) treated mice 4 d after osmotic minipump implantation. **a** BioVenn diagram to visualize the overlap of the comparisons ISO/PE vs Ctr, ISO vs. Ctr and ISO/PE vs. ISO, and the composition of the two analyzed subgroups labeled “ISO and ISO/PE matched” indicating similar transcriptional upregulation in both comparisons ISO and ISO/PE vs. Ctr and “ISO/PE combined” indicating significantly higher gene expression compared to control, control and ISO, or the ISO-treated group. **b, c** GO terms identified by pathway enrichment analysis of the indicated subgroups using Metascape software. **d** Hierarchically clustered heatmap of a curated gene list taken from the indicated GO terms summarized as extracellular matrix (ECM) organization, ECM interaction and fiber formation using Morpheus software. **e** Curated list of transcription factor motifs based on the 76 genes depicted in the heatmap and analyzed with Homer software

For ventricular transcriptome analysis, the RNAseq data sets were divided into 3 groups: genes that were significantly (*q* value < 0.05) regulated under ISO/PE vs. Ctr, ISO vs. Ctr, and ISO/PE vs. ISO. The samples were subjected to principle component analysis (PCA) to confirm correlation within the respective experimental setting (Suppl. Fig. 4), and the groups were compared via BioVenn [27] to visualize overlaps and distinctions. ISO/PE hearts showed the highest number of upregulated genes compared to control (2249), followed by ISO vs. control (1403), and ISO/PE vs. ISO (594; Fig. 6A, Suppl. Fig. 5). Pathway enrichment analysis revealed that a large part of genes which were comparably increased in both groups ISO and ISO/PE (975) are involved in cell cycle regulation (*Mki67, Cdk1, Ccnb1*) and inflammatory responses, indicating that these processes are mainly ISO-dependent (Fig. 6b).

To visualize the specific impact of additional PE stimulation, genes significantly upregulated under ISO/PE treatment either vs. control (734) or vs. both ISO and control (266), or significantly higher upregulated under ISO/PE compared to ISO stimulation (265) were combined for the analysis. Most identified GO terms were associated with the closely interconnected processes of ECM organization, cell adhesion, and fiber organization (Fig. 6c, d; Suppl Fig. 6). This group comprised genes encoding for different collagens (*Col*), ECM modulators (*Loxl1/3/4*, *Mmp14*, *Adam8/9*), the pro-fibrotic master regulator *Sox9* [55], integrins (*Itg*), structural elements (*Actb*, *Actn1/4*, *Acta2, Cfl1*, Zyx, *Flna*, *Myh9, Myo1e*) and regulators of the actin cytoskeleton (*Dock2*, *Tiam1*, *Vav1, Rhod*). In contrast to ISO alone, ISO/PE treatment also significantly induced the upregulation of genes allocated to structural remodeling of cardiac actin fibers (*Pdlim5, Wdr1, Xirp2, Ankrd23*), but not yet *Acta1* or *Myh7* (Fig. 5d). In addition, ISO/PE further promoted pro-inflammatory and pro-apoptotic genes and led to an enrichment of kinase-dependent gene transcription including ERK1/2, JNK, p38, and TGFβ associated GO terms. Consistently, increased MAPK and RhoGTPase activity under ISO/PE challenge was also reflected by the enriched motifs of transcription factors fos, c-jun, GATA, Tead2, and SRF (Fig. 6e).

### ISO/PE promotes the downregulation of genes associated with oxidative energy generation, contractility, and cardiac action potential

**Fig. 7:**
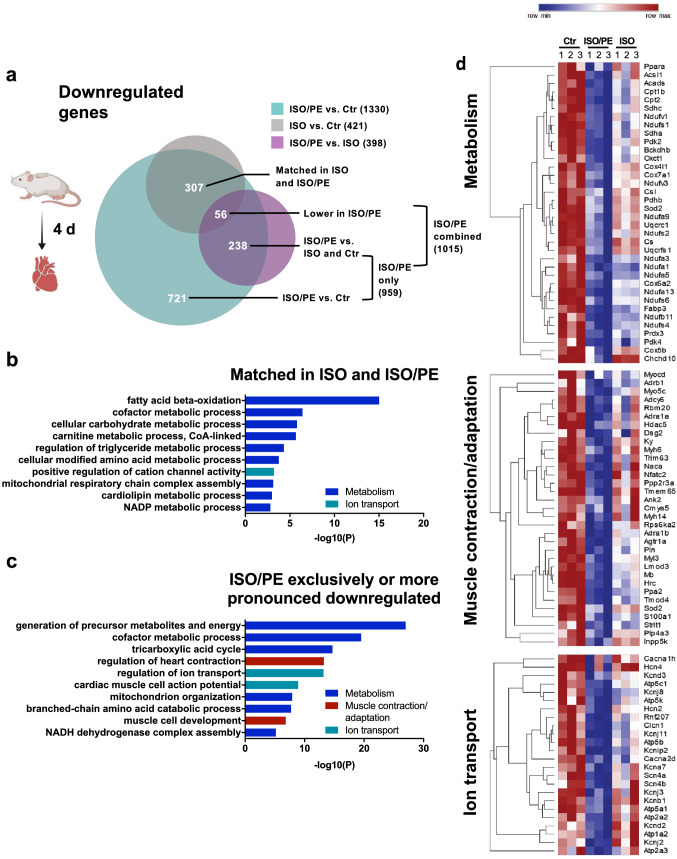
Downregulated genes (log2FC < − 0.6, *q* value < 0.05) assessed by bulk RNAseq analysis of cardiac tissue from control (0 d), isoprenaline (ISO) and isoprenaline/phenylephrine (ISO/PE) treated mice 4 d after osmotic minipump implantation. **a** BioVenn diagram to visualize the overlap of the comparisons ISO/PE vs Ctr, ISO vs. Ctr and ISO/PE vs. ISO, and the composition of the two analyzed subgroups labeled “ISO and ISO/PE matched” indicating similar transcriptional downregulation in both comparisons ISO and ISO/PE vs. Ctr and “ISO/PE combined” indicating significantly lower gene expression compared to control, control and ISO, or the ISO-treated group. **b, c** GO terms identified by pathway enrichment analysis of the indicated subgroups using Metascape software. **d** Hierarchical clustered heatmap of a curated gene list taken from the indicated GO terms summarized as metabolism, muscle contraction and adaptation, and ion transport using Morpheus software

ISO/PE treatment had also a more pronounced effect on transcriptional downregulation, exceeding ISO affected genes by threefold (1330 vs. 421, respectively, Fig. 7a). A major part of genes negatively regulated under both ISO and ISO/PE perfusion encoded components of fatty acid metabolism (Fig. 7b), comprising transporters (*Cd36, Slc27a, Cpt1a*) as well as enzymes involved in mitochondrial fatty acid degradation (*Acadl, Acadm, Eci1, Hadha, Hadhb, Ech1, Mlycd*). The switch from fatty acids as an energy substrate to glucose metabolism is a typical feature of pathological hypertrophy and described for heart failure patients [19] as well as numerous cardiac disease models including chronic ISO exposure [24].

The genes affected by additional PE treatment could be clustered into three groups: metabolism, muscle contraction and adaptation, and ion transport (Fig. 7c, d; Suppl. Fig. 7). ISO/PE perfusion led to more pronounced transcriptional alterations in metabolism by downregulating genes coding for enzymes involved in branched amino acid catabolism (*Bckdhb*), oxidative energy generation i.e. enzymes of the tricarboxylic acid cycle (*Pdh, Cs, Csl*), and mitochondrial respiratory chain (*Sdh, Nudf, Cox, Uqcrc1*), and hence to a profile rather associated with progressive HF than compensation [19, 33]. Likewise, genes involved in contraction and muscle development were stronger affected under ISO/PE treatment than under ISO alone (Fig. 7d, middle). Apart from structural components (*Myh6, Trim63, Myl3, Lmod3, Tmod4*), this group contained genes involved in transcription and RNA processing (*Myocd, Nfatc2, Cmya5, Rbm20*), conduction (*Dsg2, Ank2*), and Ca^2+^-handling (*Pln, Hrc, S100a1, Strit1*). Finally, the group summarized as “ion channels” covers genes involved in cardiac action potential (*Scn4a/b, Cacnc2d, Atp1a2, Kcnj2*) and Ca^2+^-reuptake (*Atp2a2, Atp2a3*), pacemaker activity (*Cacn1h, Hcn4, Kcnd3, Hcn2*), and channel-associated proteins (*Kcnip, Rnf207*). Another subset within this group comprised mitochondrial ATPases, which act as proton pumps (*Atp5c1, Atp5k, Atp5b, Atp5a1*). Thus, compared to exclusive ISO stimulation, ISO/PE treatment more effectively recapitulated on a transcriptional level stress-induced metabolic alterations observed in failing hearts and consequent responses that are assumed to follow energy deprivation such as the activation of the fetal gene program [62].

### Higher overlap between ISO/PE transcriptome and transcriptional alterations after TAC

To put the transcriptional characteristics of the ISO/PE and ISO models into perspective, we compared both adrenergic stress models to a mouse model of cardiac remodeling induced by transversal aortic constriction (TAC). This surgical model has similar neurohumoral features such as high SNS and RAAS activity, but with an additional mechanical stress due to pressure overload which was not observed under either ISO or ISO/PE conditions. To this end we used a published bulk transcriptome analysis derived from cardiomyocyte enriched fractions of sham and TAC-operated mice, 3 and 7 d post-surgery [49], and fused the genes that were regulated at the two time points to a single data set.

**Fig. 8:**
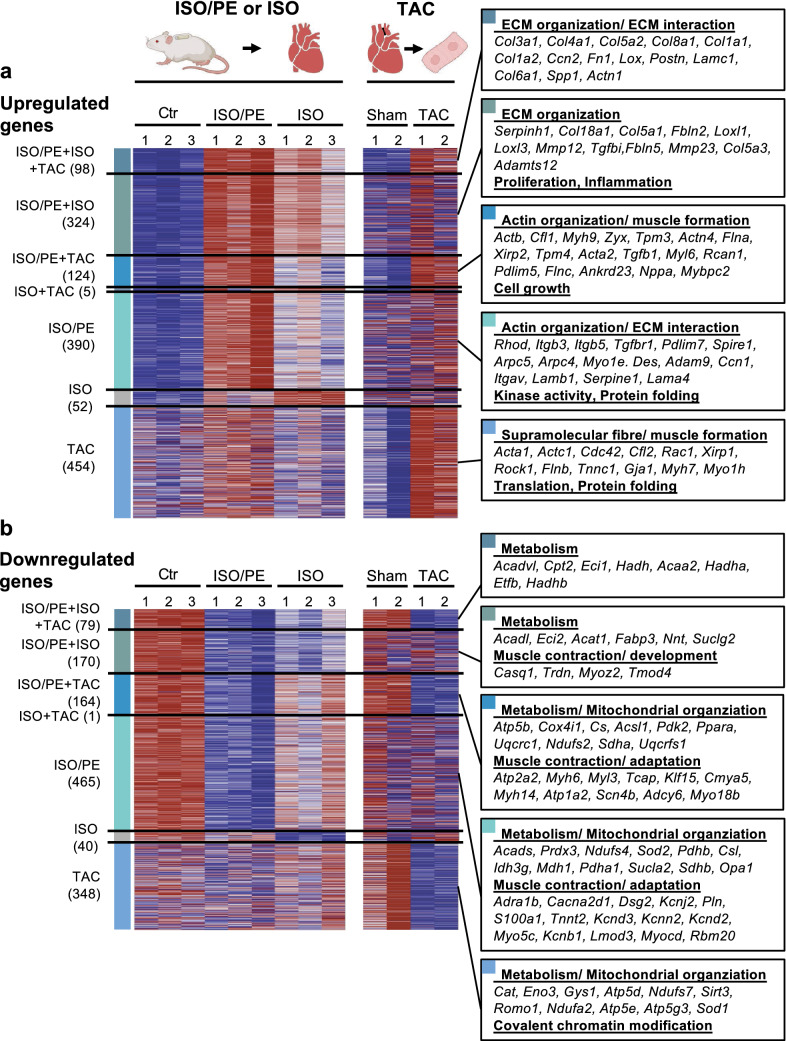
Comparison of isoprenaline (ISO) and isoprenaline/phenylephrine (ISO/PE)-induced transcriptome changes with RNAseq data from mice during the first week after transverse aortic contriction (TAC) including **a** upregulated (log2FC > 0.6; *q* value < 0.05 or *p* value < 0.05 for TAC), and **b** downregulated (log2FC < − 0.6, *q* value < 0.05 *p* value < 0.05 for TAC) genes assessed by bulk RNAseq analysis of cardiac tissue from ISO or ISO/PE-treated mice 4 d after osmotic minipump implantation, and cardiomyocyte enriched fractions taken from mice after 3 and 7 d of transaortic constriction (TAC). GO terms and representative genes were identified by pathway enrichment analysis using Metascape software

In terms of gene upregulation, ISO/PE and ISO treatment showed an overlap of 33% (222/681) and 14% (103/681) with TAC-operated mice, respectively (Fig. 8a). The GO terms included actin organization/ muscle formation, and ECM organization/ ECM interaction. Of note, 40% of the overlap between ISO/PE, ISO, and TAC (98 genes) contained the subset of genes that was significantly higher upregulated in response to ISO/PE compared to ISO treatment alone such as different collagens, *Postn*, *Spp1*, and *Ccn2*. However, despite allocated to the same pathways, not all genes enriched upon ISO/PE or ISO perfusion were also upregulated under TAC including different integrins, laminins, collagens, and genes involved in actin polymerization and branching. Conversely, TAC surgery induced a set of genes that was either not, or not yet (*Acta1, Myh7*) affect by 4 d of ISO/PE perfusion. Similarly, both TAC and ISO/PE-induced genes involved in protein folding but without a relevant overlap (Fig. 8a).

The transcriptome similarities between ISO/PE and TAC were also more pronounced regarding downregulated genes with an overlap of 41% (243/592) (Fig. 8b). In contrast, the ISO and TAC overlap remained at 13% (79/592), among those, 25% belonged to the subset of genes involved in fatty acid beta oxidation that were stronger affected under ISO/PE than under ISO treatment. The main overlapping GO terms included metabolism/ mitochondrial organization, and muscle contraction. ISO/PE and TAC shared similar GO terms regarding downregulation of genes involved in oxidative energy generation and muscle fiber remodeling, but, as demonstrated for the upregulated genes, they also showed a distinct pattern in enrichment composition. However, some of the genes that were regulated under ISO/PE but not under TAC (at the studied time points) are known factors of maladaptive cardiac remodeling such as the transcription factor myocardin (*Myocd*) [76], the Ca^2+^-binding protein S100a1 [53], and the RNA splicing factor RBM20 [22].

### Higher overlap between ISO/PE transcriptome and transcriptional alterations in human hypertrophic cardiomyopathy

**Fig. 9:**
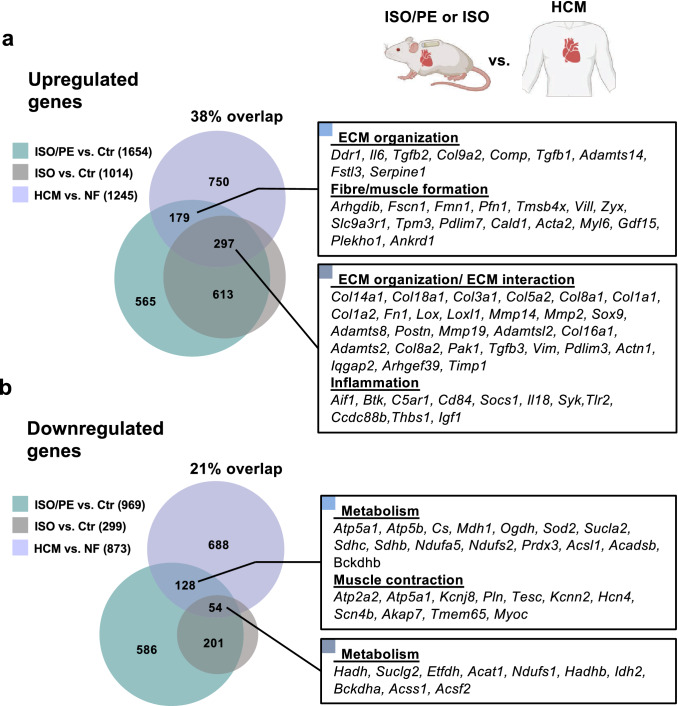
Comparison of isoprenaline (ISO) and isoprenaline/phenylephrine (ISO/PE)-induced transcriptome changes with RNAseq data from patients with hypertrophic cardiomyopathy (HCM) including **a** upregulated (log2FC > 0.6, *q* value < 0.05), and **b** downregulated (log2FC < − 0.6; *q* value < 0.05) genes assessed by bulk RNAseq analysis of cardiac tissue from ISO or ISO/PE-treated mice 4 d after osmotic minipump implantation, and 7 patients diagnosed with hypertrophic cardiomyopathy (HCM). GO terms were identified by pathway enrichment analysis using Metascape software

Finally, we compared both the ISO/PE and ISO model to a bulk transcriptome analysis of whole heart tissue from patients with hypertrophic cardiomyopathy (HCM) which was recently published by Ren et al. [54]. Regarding upregulated genes, ISO/PE treatment showed a total overlap of 38% comprising GO terms associated with ECM organization and ECM interaction, inflammation, and actin fiber/muscle formation (Fig. 9a). Interestingly, in contrast to the comparison to TAC surgery, ISO alone already covered about 60% of these overlapping genes. Among those, 33% belonged to the subset of genes that were higher upregulated under ISO/PE including different collagens and other ECM components (*Postn, Tnc, Fn1, Spp1*), the transcription factor *Sox9*, and ECM modulators (*Lox1/2/4, Mmp14, Adam12, Timp1, Cthrc1, Tgfb3*). The other 40% of the overlap derived from an ISO/PE-induced upregulation of pro-fibrotic and inflammatory genes such as cytokines (*Tgfb1/2*), and the impact on genes associated with actin bundle organization (*Fscn1, Vill*), actin polymerization (*Fmn1, Pfn1, Tmsbx4*), actin stability (*Tpm3*), and non-sarcomere actin contraction (*Cald1, Myl6*).

In contrast, the overlap of downregulated genes was lower between the ISO/PE model and HCM patients (21%, Fig. 9b). This could be at least in part attributed to the fact that the patients did not show any relevant alterations in genes involved in fatty acid beta oxidation, which was a major component regarding metabolic adaptation of all experimental models including TAC. Consistently, 70% of the overlap contained ISO/PE regulated genes which were mainly involved in other metabolic pathways such as cellular respiration (*Ndufs2/5, Sucla2, Atp5a*), TCA cycle processes (*Sdhb/c, Ogdh, Gpt*), and cardiac contraction (*Atp2a2, Pln*).

In summary, 4 d of ISO/PE challenge more profoundly reflected key transcriptome features of human HCM including pro-fibrotic modulations, inflammation, and metabolism.

## Discussion

In this explorative study, we compared combined α_1_/β-adrenergic (ISO/PE) with exclusive β-AR (ISO) stimulation to investigate the additional effect of α_1_-AR activation on early cardiac functional, structural and transcriptional adjustments to chronic catecholamine stress. Despite the presence of vasoconstrictor PE, both ISO and ISO/PE stimulation equally caused hemodynamic changes such as an increase in heart rate and a RAAS-mediated increase in blood pressure. During β_1_-AR desensitization, ISO/PE but not ISO-treated animals partially re-established acute adrenergic responsiveness due to an increased contribution of α_1_-AR-mediated inotropy. ISO/PE-induced less concentric growth of cardiomyocytes which was accompanied by increased collagen deposition and more pronounced activation of genes related to ECM and actin fiber composition. Additional PE treatment further caused more profound alterations in transcriptional patterns comprising genes involved in metabolism and cardiac ion homeostasis. Consistently, transcriptome changes under ISO/PE challenge showed a higher overlap with an experimental model of pressure overload-induced cardiac remodeling and human hypertrophic cardiomyopathy than exclusive ISO stimulation, indicating a more effective recapitulation of the complex neurohumoral activation preceding the development of heart failure.

### Impact of RAAS activity and hemodynamics

Besides chronic elevation of catecholamines, increase in Ang II levels due to RAAS hyperactivity is another major driver of cardiac remodeling. RAAS activation is triggered when blood pressure/volume is insufficient to maintain organ perfusion, and by renal β-AR activity. Consistently, genes involved in AngII/TGFβ-induced remodeling processes were found to be upregulated under both chronic ISO and ISO/PE challenge, with higher impact of ISO/PE especially on pro-fibrotic gene expression. Despite the vasoconstrictive effects of PE, differences in BP regulation under ISO/PE stimulation compared with ISO alone were not observed and both models displayed an AT_1_R-dependent hemodynamic compensation of β-AR-mediated vasodilatation, suggesting similar systemic RAAS activation. In total, these findings argue against additional relevant PE-induced hemodynamic differences in the presence of ISO. Another potent trigger of fibroblast activation and hypertrophic growth is mechanotransduction [39]. Although α_1A_-AR-mediated contractility may add to mechanical strain of the heart [6], it remained part of a maximal stress response, whereas basal cardiac contractility in vivo was similarly elevated under both chronic ISO and ISO/PE perfusion. With hypertension basically absent and fibrosis not yet developed, the mechanical strain in the early stage of the ISO/PE model therefore seemed to mainly derive from ISO-induced increases in heart rate and contraction force and at that point is assumed to be similar in both adrenergic models. Thus, we conclude that neither transcriptional modulations nor the limited concentric growth observed under ISO/PE conditions was based on differences in systemic RAAS activity, hemodynamic alterations, or mechanical strain. This finding is therefore in line with the use of PE as an additional, and independent stressor during chronic Ang II perfusion [9].

### AR expression levels and inotropic responsiveness of α_1_-AR

Our data demonstrate that ISO/PE but not ISO challenged mouse hearts gained the capacity of acute adrenergic responsiveness through α_1_-AR-mediated positive inotropy, thus reflecting the described increase in contribution of α_1_-ARs to cardiac pump function during β_1_-AR desensitization and in human HF patients [16, 29, 58]. Pro-inotropic effects occurred despite the downregulation of α_1A/B_-AR mRNA levels, indicating a sufficient α_1A/B_-AR receptor reserve in mice. Interestingly, reduction in α_1_-AR mRNA expression was either similarly (α_1A/B_-AR) or exclusively (α_1D_-AR) affected by ISO alone, substantiating the concept that unlike β_1/3_-ARs, α_1_-AR expression levels might be independent of chronic agonist exposure [35]. Of note, while meanBP was similar in both models, lower expression of cardiac α_1D_-AR mRNA under ISO conditions may suggest that coronary blood flow could be differently affected [68], although the functional consequences of this reduction are yet to be further investigated.

α_1_-AR-mediated contraction is associated with both modulation in Ca^2+^-sensitivity and Ca^2+^-transients [16]. Over the last decades numerous targets were identified such as the Na^+^/H^+^-exchanger [60], myosin light chain kinase [1, 8, 21, 72, 79], the L-type Ca^2+^ channel [50], and TRPC6 [43]. While at the early stage of cardiac adaptation none of these specified components were (yet) directly affected on a transcriptional level (i.e., *Mylk, Slc9a1*, *Rock1*), ISO/PE perfusion, either exclusively or more pronounced, induced a multitude of transcriptional alterations regarding genes involved in sarcomere structure and function, thereby most likely facilitating the positive inotropic response to α_1_-AR stimulation. Although the underlying mechanisms of α_1_-AR-mediated contractility and their proposed protective effects require further investigation in this model, chronic ISO/PE challenge seems to be a useful setting to capture the reported increase in α_1_-AR-mediated left ventricular inotropy during β_1_-AR (and possibly β_3_-AR) desensitization in mice.

### Transcriptional and morphological stress adaptations

**Fig. 10:**
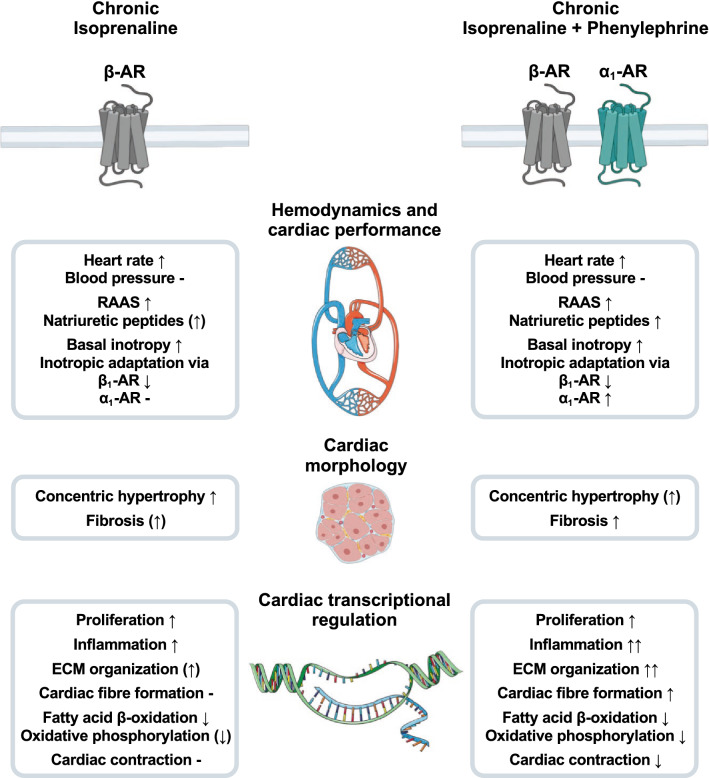
Schematic overview of the comparison between exclusive isoprenaline and combined isoprenaline + phenylephrine stimulation during the first 7 d of chronic perfusion. *RAAS* renin–angiotensin–aldosterone system, *AR* adrenoceptor, *ECM* extracellular matrix

Both ISO and ISO/PE perfusion led to an array of transcriptional changes including genes involved in processes such as ECM remodeling and interaction, inflammation, proliferation, apoptosis, reactive oxygen stress responses, metabolism, contractility, and conduction; all relevant features of deteriorating cardiac function [2, 7, 19, 45, 69]. The most prominent impact of additional PE stimulation centered on ECM organization, muscle or cytoskeletal fiber formation, oxidative phosphorylation, and conduction. While α_1_-AR-dependent activation of adaptational responses such as the re-expression of fetal genes e.g., involved in sarcomere structure and function (*Acta1*, *Myh7*, *Xirp2*, *Pdlim5*) is a long-standing concept, α_1_-ARs have been reported to mediate a much more global impact on cardiac stress responses including non-cardiomyocytes. This is especially interesting because apart from smooth muscle cells, α_1_-ARs were mostly reported absent in other non-cardiomyocyte cell populations [35]. However, cardiomyocyte-specific α_1A_-AR overexpression was shown to drive progressive fibrosis and reactivation of genes encoding matricellular proteins in the absence of pharmacological or surgical interventions [6], and angiogenesis after myocardial infarction [80], indicating a potential role for α_1_-AR signaling in cell–cell communication and organ homeostasis. If the early increase in fibroblast response in this study will also manifest in a more severe cardiac fibrosis under long-term ISO/PE challenge or lack relevant fibrotic cardiac remodeling as often reported from the ISO model [15, 17, 74] has yet to be investigated. Under both ISO and ISO/PE treatment, activation of fibroblast associated genes (*Tgfb1, Postn, Col1a1*) was no longer observed at 7 d of exposure, and thus might rather reflect the previously reported timely correlation between gene programs involved in fibroblast activity and hypertrophic growth than the onset of fibrosis [11, 54, 63]. Another notable feature of the ISO/PE model is the impact on genes involved in metabolic processes. In diseased and failing hearts metabolism is shifted to more glucose utilization for efficient energy production, while mitochondrial oxidation and electron transport chain activity are compromised [33]. Whereas reduction in fatty acid utilization is associated with chronic ISO exposure, ISO/PE stimulation had a more prominent impact on genes involved in oxidative phosphorylation and mitochondrial organization, and thus showed a higher overlap in transcriptional patterns with both experimental TAC and human HCM. To date, there is little known about α_1_-AR-mediated regulation of cardiac metabolism. In murine hearts, α_1_-AR activation is associated with increased glucose uptake, which may facilitate the switch in substrate utilization during cardiac stress conditions [57]. Together with the more pronounced impact on genes involved in cardiac contraction, combined α_1_/β-stimulation seems at least in this early stage to evoke compensatory responses to sustained adrenergic stress more efficiently, which may therefore explain the earlier termination of concentric hypertrophic growth in the ISO/PE model. Chronic α_1_-AR stimulation thus is an essential component of the adaptive as well as maladaptive processes associated with the development of heart failure (Fig. 10).

### Limitations of the ISO/PE model and technical limitations

Pharmacological models such as ISO/PE can only partially reflect endogenous sympathetic activation. They lack the effects of released co-transmitters such as neuropeptide Y [64] and in case of ISO/PE, the impact on the coronary microvasculature where α_2_-ARs predominate [25, 26]. In addition, differences between species regarding the distribution, densitiy and function of adrenergic receptors may have to be considered [4, 35]. Lastly, the α_1_-AR agonist PE also shows affinity to β_2_-AR, but with fourtyfold higher EC_50_ values than ISO [52]. Since both substances were given in equal doses, we think it is highly unlikely that the β_2_-AR affinity of PE has a relevant impact in this model but cannot completely rule out that some effects are related to higher β_2_-AR and not α_1_-AR activity.

A major concern of bulk RNAseq data sets from whole cardiac tissue is that the downregulation of cardiomyocyte-specific genes might be overrepresented and at least partially derive from a shift towards non-cardiomyocyte gene expression. However, there is a high overlap between the respective GO terms enriched in the ISO/PE data set and the TAC data set which contained only mRNA from isolated cardiomyocytes and therefore a minimized dilution by non-cardiomyocytes. In addition, multiple cardiomyocyte marker genes such as *Ryr2* and *Ttn* were unchanged in the ISO and ISO/PE group vs. control, indicating no substantial change in the proportion of cardiomyocyte vs. non-cardiomyocyte gene expression in the heart at the time point used for RNAseq analysis. For higher transparency, we exemplarily visualized the normalized reads of cardiomyocyte and fibroblast marker genes, and regulated genes from the major pathways in Suppl. Figs. 6 and 7.

Finally, since this study focused on early events of chronic catecholamine challenge, the manifestation of ISO/PE-induced heart failure and associated transcriptional alterations remains yet to be investigated.

## Conclusion

Neurohumoral interventions are easy to apply with a low inter-individual variability in functional and molecular responses but may lack some characteristics expected from a cardiac disease model. α_1_/β-adrenergic activation via chronic ISO/PE perfusion delivers critical characteristic features of pathological hypertrophy and adaptational stress responses that are absent or less pronounced under exclusive β-AR stimulation, without differentially interfering with the essential impact of RAAS activity on cardiac remodeling processes. Despite the absence of pressure overload, the ISO/PE model therefore seems in many regards a more suitable intervention to analyze the pleiotropy of molecular and structural changes associated with chronic sympathetic overdrive that are preceding the development heart failure.

## Supplementary Information

Below is the link to the electronic supplementary material.

Supplementary file1 (PDF 5505 KB)
