# Supplementary material for: Chronic isoprenaline/phenylephrine vs. exclusive isoprenaline stimulation in mice: critical contribution of alpha1-adrenoceptors to early cardiac stress responses
Source: Basic Res Cardiol. 2022 Mar 14;117(1):15. doi: 10.1007/s00395-022-00920-z (PMC8921177; doi:10.1007/s00395-022-00920-z)
Supplement: Supplementary file 1 — Supplementary file1 (PDF 5505 KB) [file 395_2022_920_MOESM1_ESM.pdf]

Suppl. Table 1

Parameter basal	NaCl	ISO	ISO/PE	ISO vs. NaCl p-value	ISO/PE vs. NaCl p-value	ISO/PE vs. ISO p-value
HR (bpm)	355±67	532±51	552±46	**<0,0001**	**<0,0001**	0,7989
FS (%)	32±6	50±6	48±3	**<0,0001**	**<0,0001**	0,8384
SV (µl)	47±4	50±9	38±6	0,7027	0,0658	**0,0164**
EF (%)	61±8	81±6	80±3	**<0,0001**	**<0,0001**	0,9603
CO (ml/min)	17±4	26±4	21±3	**0,0002**	0,0835	**0,0500**
LVDd (mm)	4.2±0.3	3.8±0.3	3.4±0.2	**0,0387**	**0,0004**	0,0953
LVVd (µl)	79±14	62±12	48±8	**0,0309**	**0,0004**	0,1226
LV mass (mg)	87±18	131±35	95±5	**0,0374**	0,5590	0,0862
LVAWd (mm)	0.73±0.10	1.24±0.21	1.05±0.10	**<0,0001**	**0,0023**	0,0839
LVPWd (mm)	0.67±0.13	0.90±0.19	0.88±0.10	**0,0235**	**0,0471**	0,9756

Parameter DOBU	NaCl	ISO	ISO/PE	ISO vs. NaCl p-value	ISO/PE vs. NaCl p-value	ISO/PE vs. ISO p-value
HR (bpm)	473±51	533±40	543±37	**0,0391**	**0,0144**	0,8917
FS (%)	59±9	49±7	58±6	0,0597	0,9733	0,1233
SV (µl)	46±5	52±9	37±7	0,2028	0,0941	**0,0034**
EF (%)	75±5	80±7	84±3	0,2127	**0,0137**	0,3369
CO (ml/min)	19±5	28±4	20±4	**0,0059**	0,9218	**0,0222**
LVDd (mm)	3.8±0.2	3.9±0.4	3.3±0.3	0,8684	**0,0077**	**0,0034**
LVVd (µl)	64±7	66±14	45±10	0,8884	**0,0084**	**0,0041**

Suppl. Table 1 Echocardiographic parameters assessed in control (NaCl), isoprenaline (ISO, 30 mg/kg*d) or isoprenaline/phenylephrine (ISO/PE, 30 mg/kg*d each) groups after 6 d of chronic perfusion. Mean ± SD of n = NaCl: 4 males/4 females, ISO: 4 males/3 females; ISO/PE: 3 males/3 females) are shown. P-values were determined by ordinary 1-way ANOVA followed by Tukey`s multiple comparison test or Brown-Forsythe and Welch ANOVA Dunnett T3 Multiple Comparison (for LV mass comparison).

Suppl. Table 2

Parameter	NaCl	ISO	ISO/PE	ISO vs. NaCl p-value	ISO/PE vs. NaCl p-value	ISO/PE vs. ISO p-value
**BW 0d (g)**	22.2±2.9	23.3±2.7	22.3±2.7	0,7559	0,9942	0,8137
**BW 7d (g)**	22.3±2.6	24.5±2.4	20.7±2.0	0,3632	0,5809	0,0596
**HW 7d (mg)**	108.9±13.3	152.0±25.5	133.4±12.4	**0,0013**	0,0788	0,2282
**Tibia length 7d (mm)**	17.0±0.5	16.8±0.6	17.4±0.3	0,7840	0,4085	0,1693

Suppl. Table 2 Biometric data assessed in control (NaCl), isoprenaline (ISO, 30 mg/kg*d) or ISO/phenylephrine (ISO/PE, 30 mg/kg*d each) groups either before (0 d) or after 7 d of chronic perfusion. Mean ± SD of n = NaCl: 4 males/4 females, ISO: 4 males/3 females; ISO/PE: 3 males/3 females) are shown. P-values were determined by 1-way ANOVA followed by Tukey`s multiple comparison test.

Suppl. Fig. 1

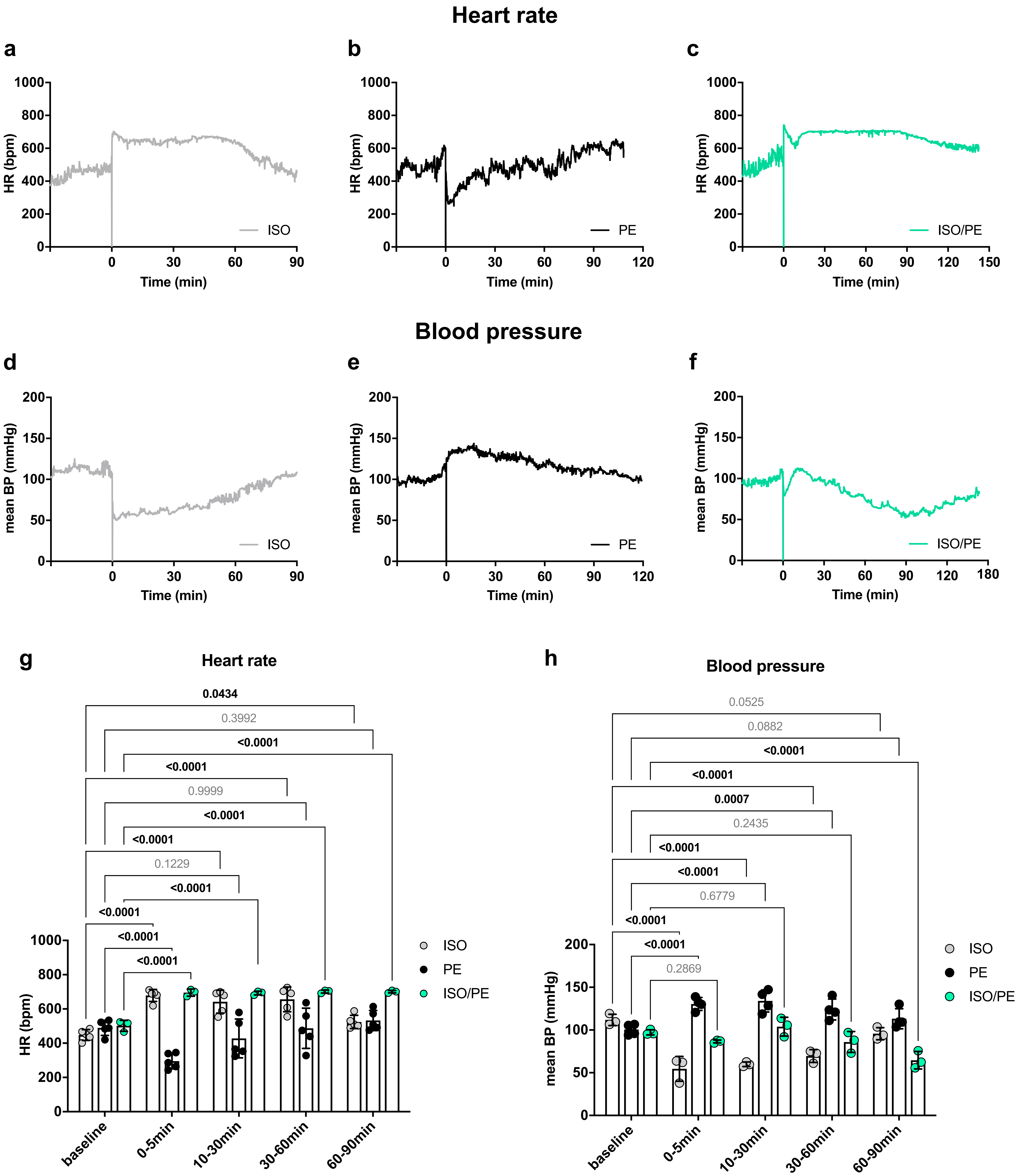

Suppl. Fig. 1 Hemodynamic profile of acute isoprenaline (ISO 3 mg/kg), phenylephrine (PE 3 mg/kg) or ISO/PE (3 mg/kg each) treated mice. **a-c** Heart rate (HR, upper panel) and **d-f** mean blood pressure (BP, lower panel) were monitored by ECG and BP telemetry in freely moving mice and presented as averages of 5 s intervals. Calculations of average values for **g** HR and **h** BP at the indicated time points. Individual data, mean ± SD of n = 3-5 male animals are shown. P-values were determined by repeated measures 2-way ANOVA followed by Dunnett`s multiple comparison test (vs. baseline).

Suppl. Fig. 2

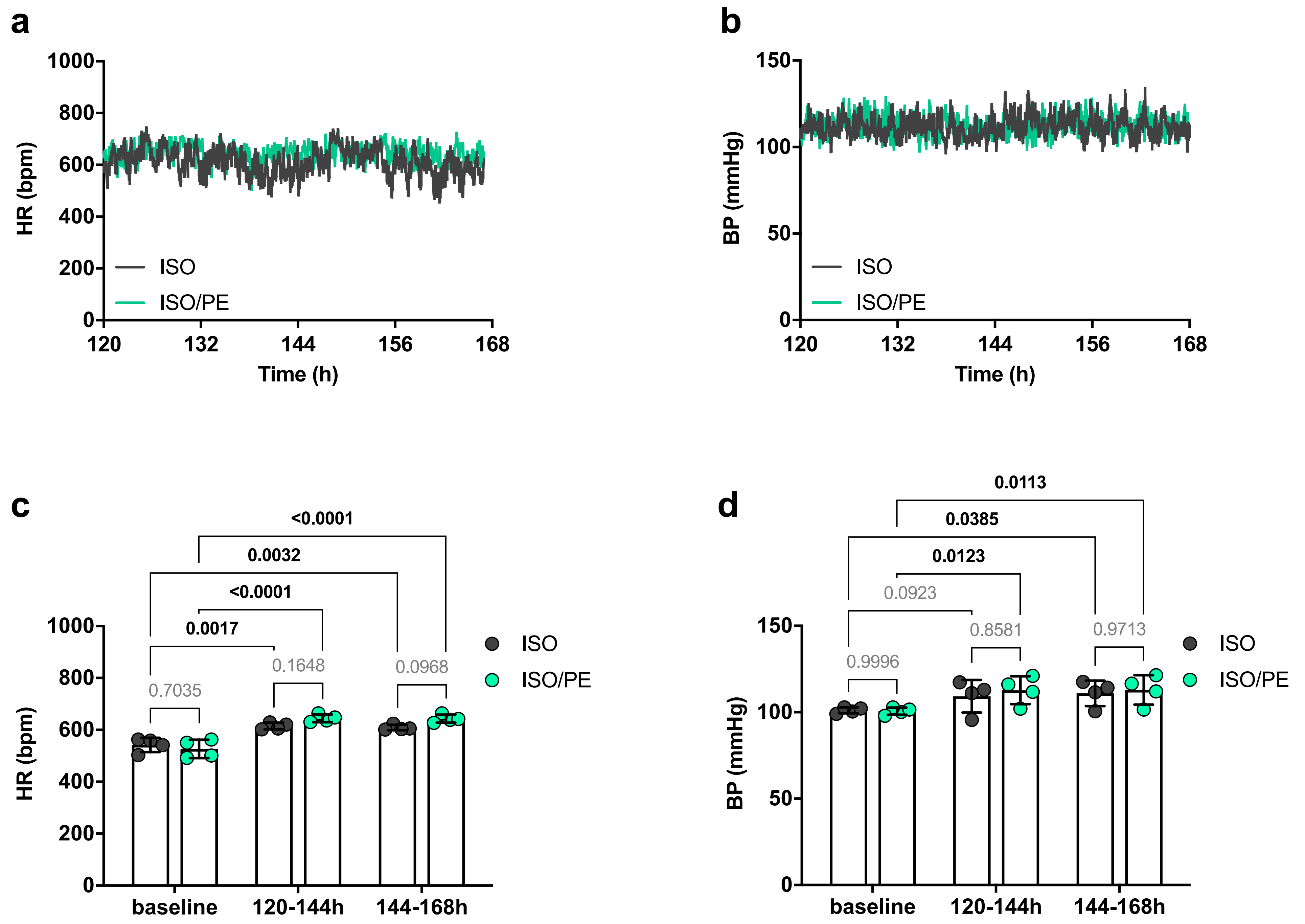

Suppl. Fig. 2 Hemodynamic profile of isoprenaline (ISO) or isoprenaline/phenylephrine (ISO/PE) treated mice between 5 d and 7 d after osmotic minipump implantation. **a** Heart rate (HR) and **b** mean blood pressure (BP) were monitored by ECG and BP telemetry in freely moving mice and presented as averages of 1 min intervals. Calculations of average values for **c** HR and **d** BP at the indicated time points are given including an average of 3 d baseline recordings. Individual data, mean ± SD of n = 4 animals (2 males and 2 females per group) are shown. P-values were determined by repeated measures 2-way ANOVA followed by Dunnett`s multiple comparison test (vs. baseline) and Sidak`s multiple comparisons of means (ISO/PE vs. ISO).

Suppl. Fig. 3

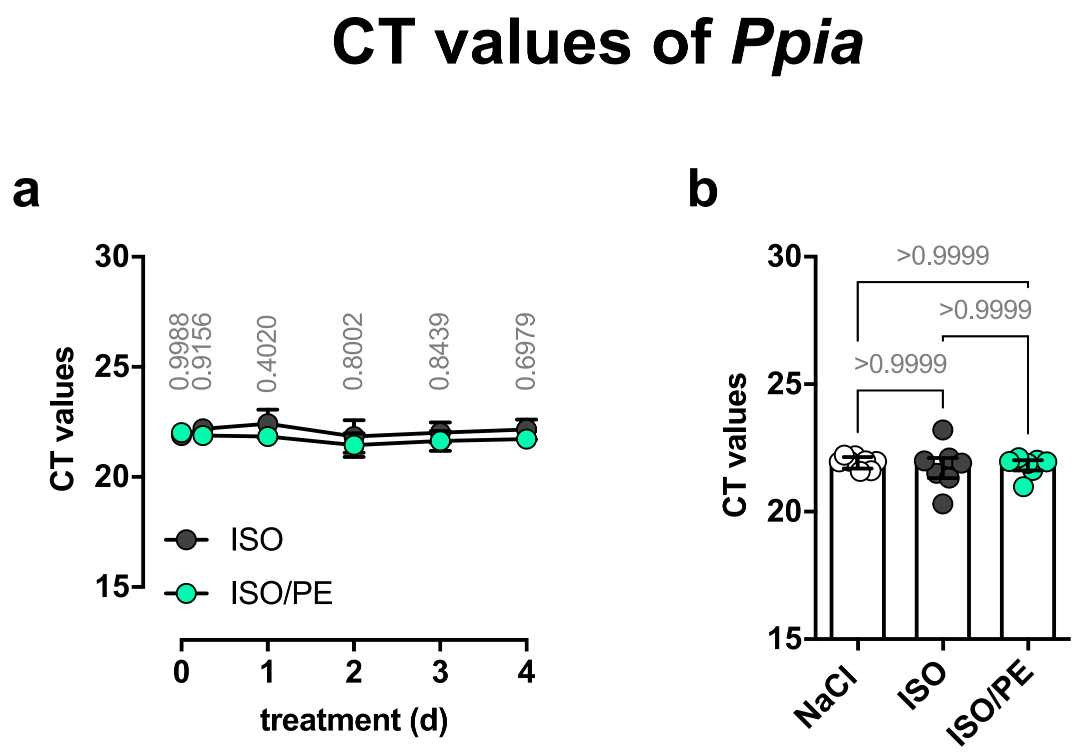

Suppl. Fig. 3 Average CT values of the reference gene peptidylprolyl isomerase A (Ppia) used for quantitative PCR during time-course experiments and after 7d of isoprenaline (ISO) or isoprenaline/phenylephrine (ISO/PE) exposure. **a** Mean ± SD, p-values were determined by 2-way ANOVA followed by Sidak`s multiple comparison of means (ISO/PE vs. ISO) for each time point, **b** Individual data, median ± IQR, p-values were determined by Kruskal-Wallis Test followed by Dunn's multiple comparison.

Suppl. Fig. 4

a

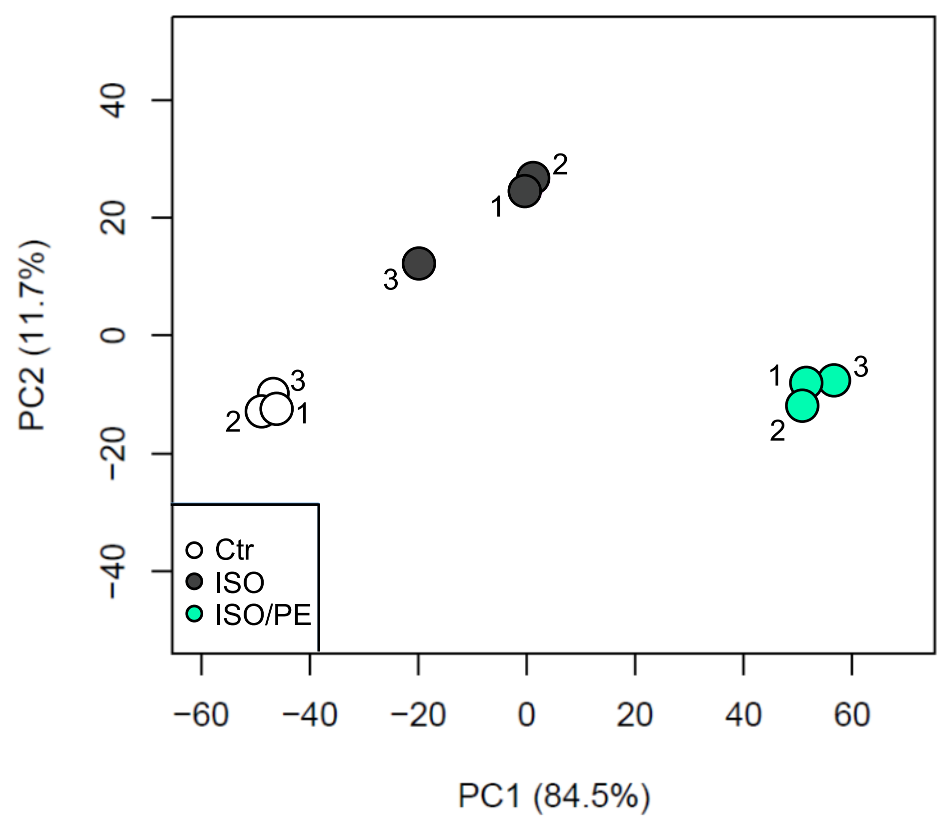

b

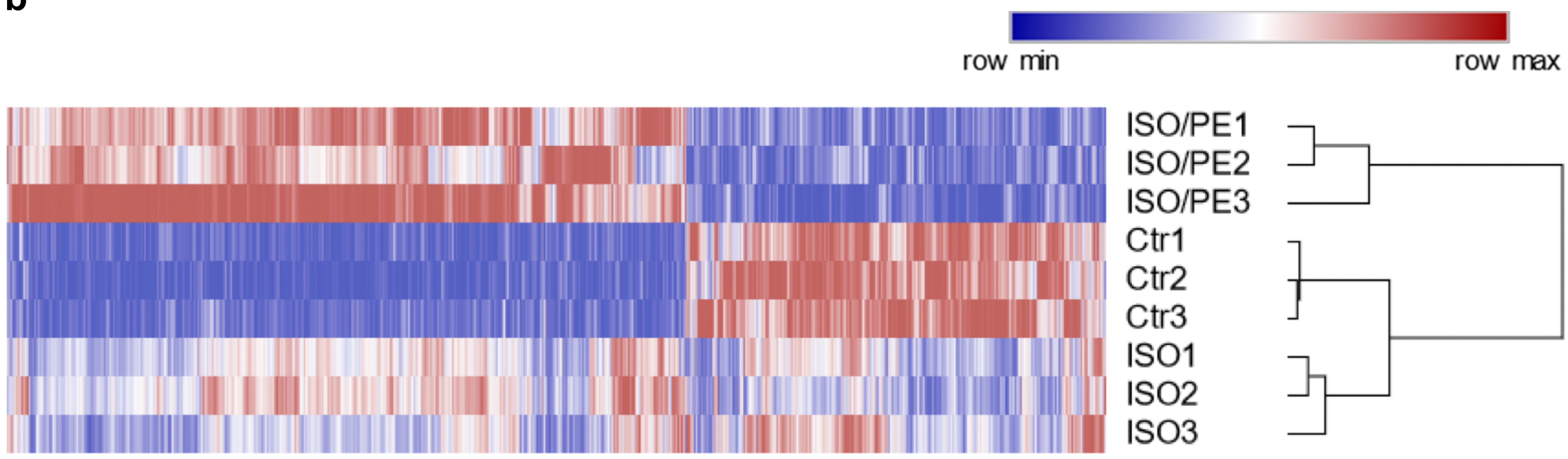

Suppl. Fig. 4 Correlation between RNAseq data sets of control animals (Ctr), and animals treated for 4 days with either isoprenaline (ISO) or isoprenaline/phenylephrine (ISO/PE). **a** Principal component analysis (PCA) of the top 1000 RPKM. **b** Heatmap and hierarchical clustering of all genes significantly regulated under ISO/PE treatment.

Suppl. Fig. 5

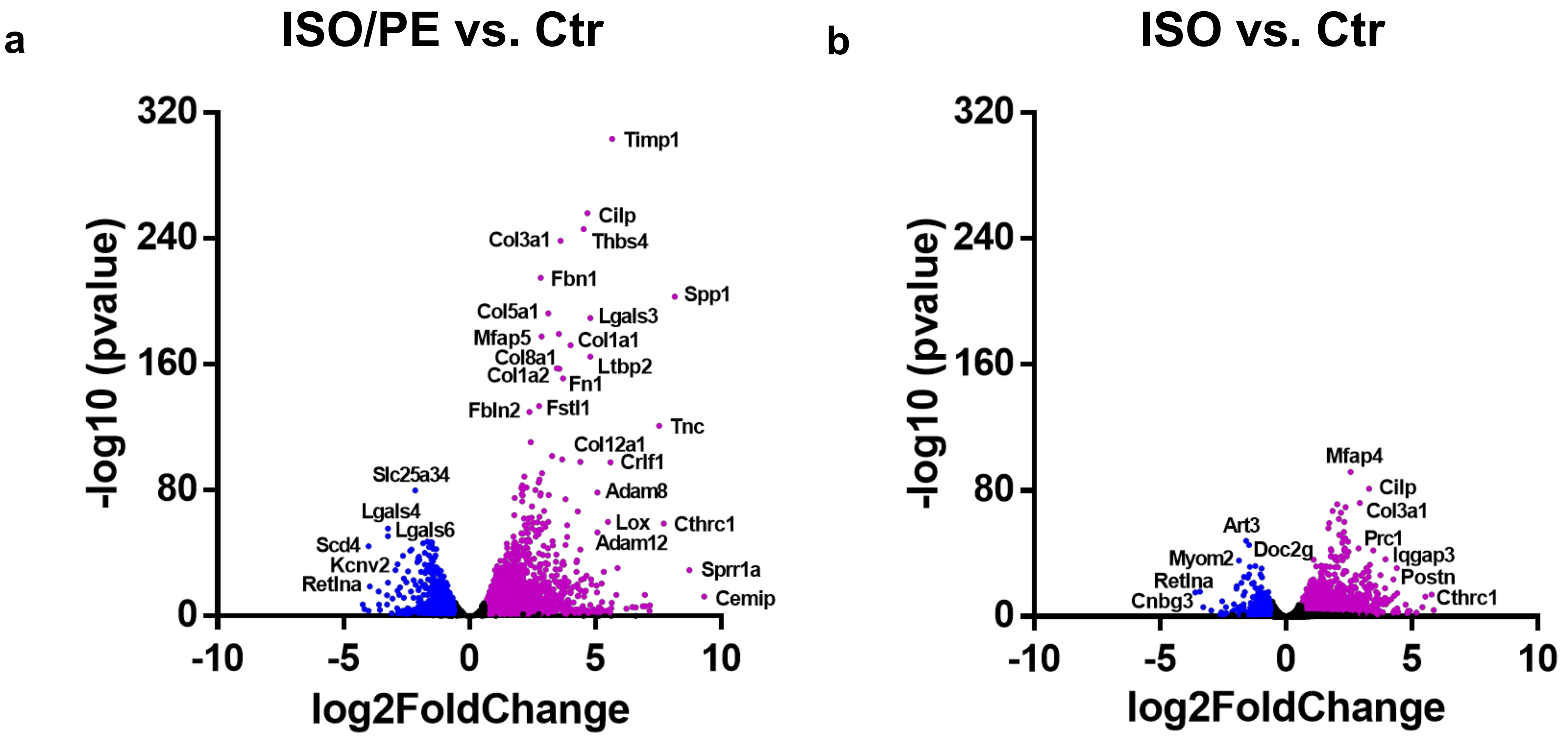

Suppl. Fig. 5 Volcano plot of > 14000 genes identified in the RNAseq data set of animals treated for 4 days with either **a** isoprenaline plus phenylephrine (ISO/PE) or **b** ISO. Upregulated gene are shown in magenta (log2FoldChange > 0.6; padj < 0.05), downregulated genes in blue (log2FoldChange < -0.6; padj < 0.05), and non-regulated genes in black. Prominently regulated genes are individually marked.

Suppl. Fig. 6

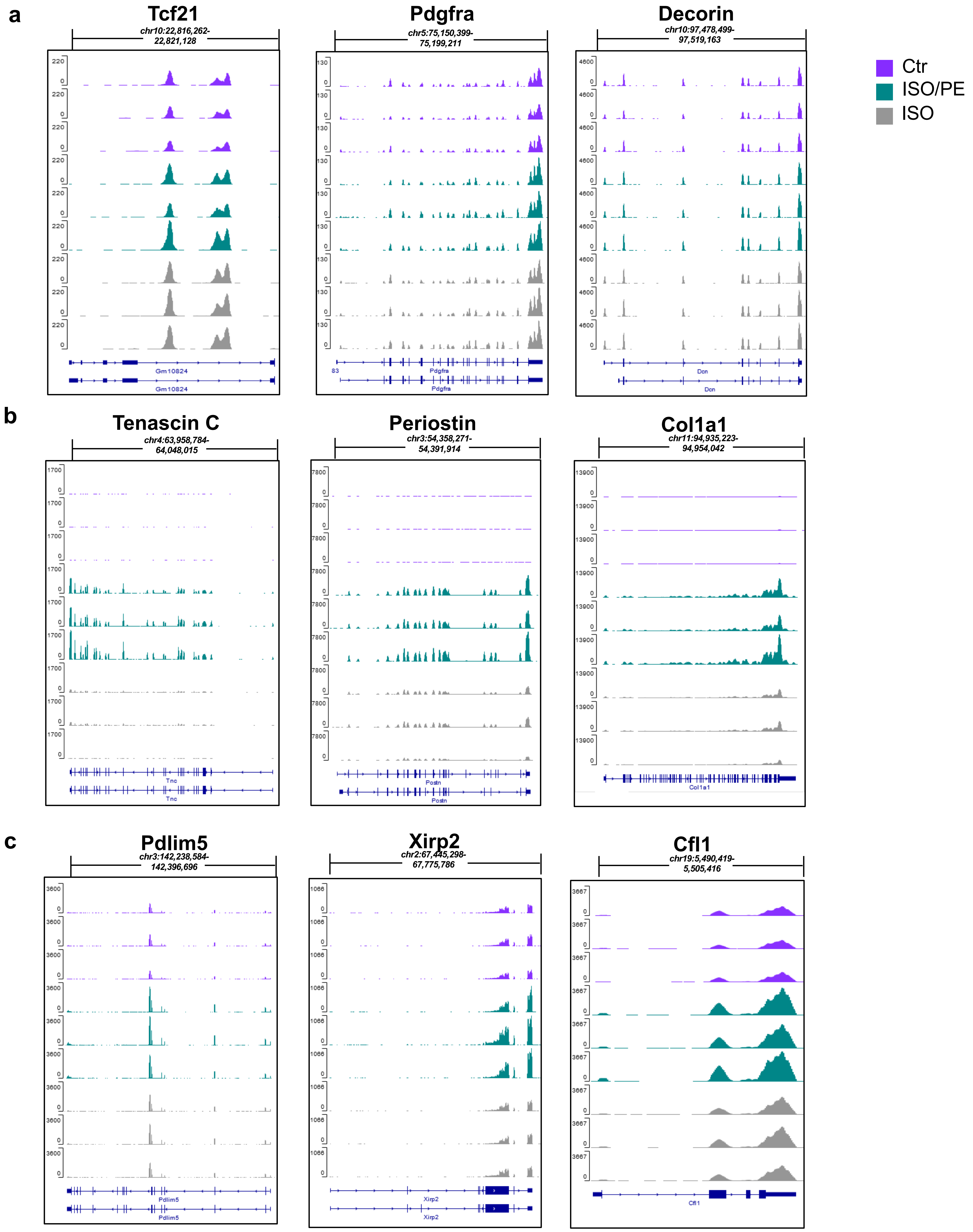

Suppl. Fig. 6 Visualization of RPKMs by Integrative Genomics Viewer. Examples of **a** fibroblast marker genes, and upregulated genes from **b** ECM related, and **c** actin fibre formation related GO terms.

Suppl. Fig. 7

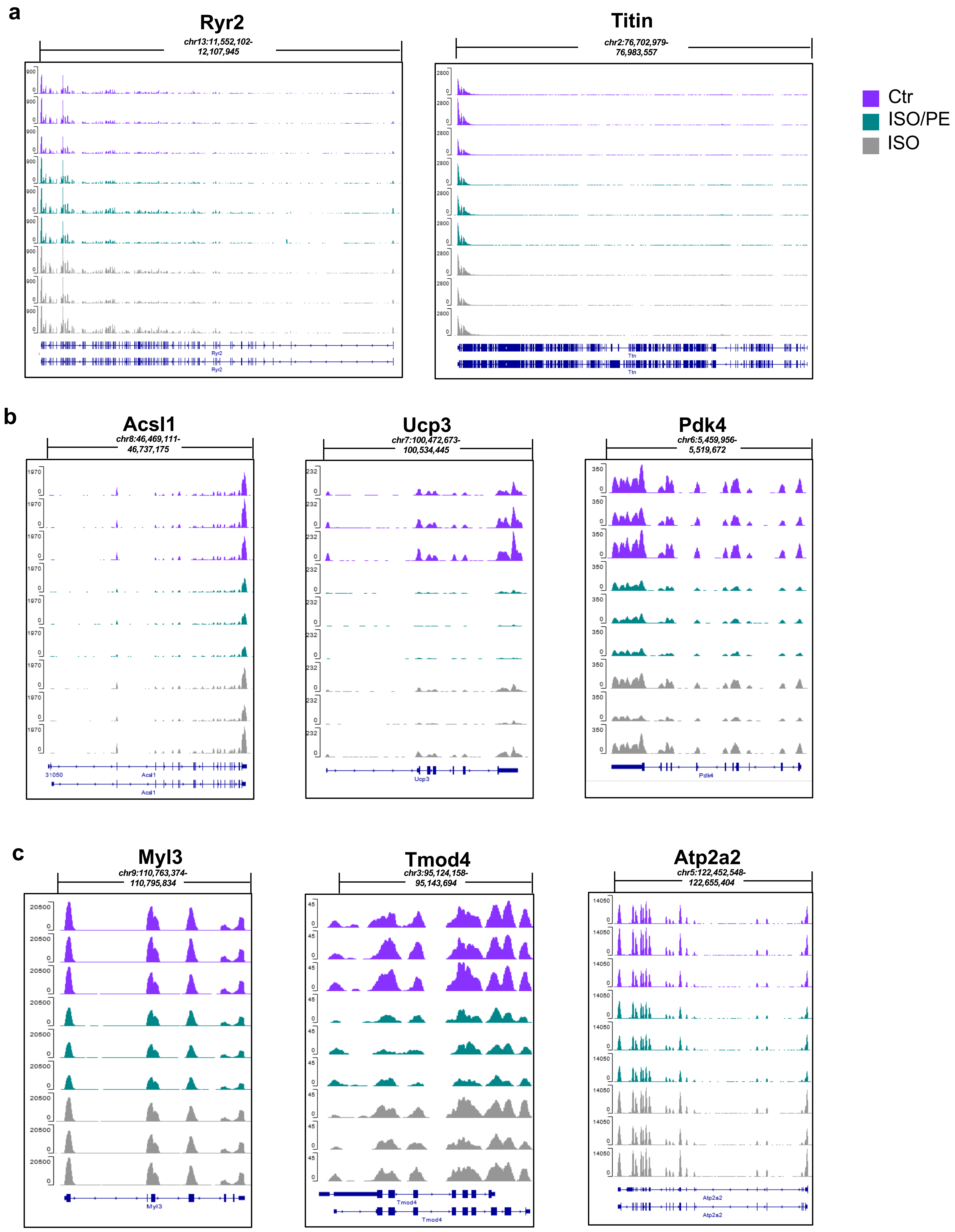

Suppl. Fig. 7 Visualization of RPKM values by Integrative Genomics Viewer. Examples of **a** stably expressed cardiomyocyte marker genes, and downregulated genes from **b** metabolic, and **c** muscle related GO terms.
